# BDNF Val66Met (rs6265) in Ankylosing Spondylitis: A Descriptive Genotype–Phenotype Study in a Romanian Cohort

**DOI:** 10.3390/cimb48101018

**Published:** 2026-10-02

**Authors:** Laura Țiburcă, Alexandru Daniel Jurca, Darius Lotrean, Romana Vulturar, Adina Chiș, Ramona Hodișan, Codruta Diana Petchesi, Emilia Severin, Claudia Maria Jurca

**Affiliations:** 1 Doctoral School of Biological and Biomedical Sciences, University of Oradea, 410087 Oradea, Romania; tiburca.lauraelena@student.uoradea.ro; 2 Department of Preclinical Disciplines, Faculty of Medicine and Pharmacy, University of Oradea, 410087 Oradea, Romania; alexjurca@uoradea.ro (A.D.J.); codruta.diana.petchesi@didactic.uoradea.ro (C.D.P.); maria.jurca@didactic.uoradea.ro (C.M.J.); 3 Faculty of Medicine, “Iuliu Hațieganu” University of Medicine and Pharmacy, 34 Victor Babeș St., 400012 Cluj-Napoca, Romania; lotrean.darius@elearn.umfcluj.ro; 4 Discipline of Cell and Molecular Biology, “Iuliu Hațieganu” University of Medicine and Pharmacy, Cluj-Napoca, Romania, 6 Pasteur St., 400349 Cluj-Napoca, Romania; romanavulturar@gmail.com (R.V.); adinachis82@gmail.com (A.C.); 5 Department of Genetics, University of Medicine and Pharmacy “Carol Davila” Bucharest, Dionisie Lupu Street, Number 37, 020021 Bucharest, Romania

**Keywords:** ankylosing spondylitis, radiographic axial spondyloarthritis, BDNF Val66Met, rs6265, genotype–phenotype association, metabolic syndrome, cardiometabolic risk, disease activity, inflammation, pain, depressive symptoms

## Abstract

**Background:** Ankylosing spondylitis (AS) is a chronic inflammatory disease with musculoskeletal and extra-articular manifestations and associated cardiometabolic comorbidity. Brain-derived neurotrophic factor (BDNF) is involved in pain processing, neuroplasticity, inflammatory signaling, and metabolic regulation. The functional BDNF Val66Met polymorphism (rs6265) affects intracellular trafficking and activity-dependent BDNF secretion, but its clinical relevance in AS remains poorly characterized. This study evaluated the distribution of BDNF Val66Met and explored its associations with disease activity, inflammation, pain, depressive symptoms, and metabolic parameters in patients with AS. **Methods:** This single-center, cross-sectional study included 81 patients with AS and no healthy control group. Genotyping was performed using a tetra-primer amplification refractory mutation system polymerase chain reaction (T-ARMS-PCR) assay. Clinical assessment included the Bath Ankylosing Spondylitis Disease Activity Index (BASDAI), Ankylosing Spondylitis Disease Activity Score based on C-reactive protein (ASDAS-CRP), pain Numerical Rating Scale (NRS), and depression subscale of the Hospital Anxiety and Depression Scale (HADS-D), together with CRP and metabolic measurements. ASDAS-CRP was designated as the primary clinical outcome. Statistical analyses included the Mann–Whitney U test, Fisher’s exact test, Spearman correlation with Benjamini–Hochberg false discovery rate correction, Hardy–Weinberg equilibrium testing, and exploratory multivariable linear and logistic regression. **Results:** The cohort comprised 81 patients (61 men and 20 women; mean age 53.2 ± 12.5 years). T-ARMS-PCR yielded 57 GG (70.4%) and 24 GA (29.6%) genotype calls, with no AA banding pattern observed; the Met allele frequency was 14.8%. The genotype calls were not independently confirmed by sequencing or restriction fragment length polymorphism analysis. No significant associations were identified between genotype and the clinical, inflammatory, or metabolic variables. Genotype was not associated with ASDAS-CRP after adjustment for age, sex, disease duration, current biologic-therapy status, and current smoking status (B = 0.045, 95% CI −0.238 to 0.328, *p* = 0.754; *n* = 80). After Benjamini–Hochberg correction, pain NRS remained correlated with BASDAI and CRP, while HADS-D remained correlated with BASDAI. Metabolic syndrome was present in 24 of 81 participants (29.6%) according to the harmonized Joint Interim Statement definition; medication use for individual metabolic components was not systematically available. **Conclusions:** No evidence of an association between BDNF Val66Met and disease activity, inflammatory markers, depressive symptoms, or metabolic parameters was found in this AS cohort. This study had 80% power to only detect an effect of Cohen’s d ≥ 0.69, and smaller genotype-associated differences cannot be excluded. The findings also describe the cardiometabolic profile of the cohort and the relationships among pain, inflammation, and depressive symptoms.

## 1. Introduction

Ankylosing spondylitis (AS) is a chronic immune-mediated inflammatory disease within the spondyloarthritis (SpA) spectrum, primarily affecting the axial skeleton [1]. Persistent sacroiliac and spinal inflammation may cause chronic pain, progressive structural damage, and, in advanced disease, ankylosis. Peripheral joints, entheses, and extra-articular organs may also be involved; common extra-articular manifestations include anterior uveitis, psoriasis, and inflammatory bowel disease [2,3].

The HLA-B27 allele is present in AS patients worldwide, with reported prevalence ranging from approximately 80% to 95% depending on ethnicity and geographic region [4]; however, genetic susceptibility extends beyond HLA-B27 and involves additional MHC and non-MHC loci [5,6]. All 81 patients in the present cohort were HLA-B27 positive (see Section 2.1). Current evidence supports an important role for the IL-23/IL-17 pathway in disease pathogenesis and maintenance of chronic inflammation [1].

Disease activity in AS is assessed using validated composite indices. The Bath Ankylosing Spondylitis Disease Activity Index (BASDAI) is a patient-reported instrument evaluating fatigue, spinal pain, peripheral joint symptoms, enthesitis, and morning stiffness, with scores ranging from 0 to 10 [7]. The Ankylosing Spondylitis Disease Activity Score (ASDAS) incorporates objective inflammatory markers such as CRP or ESR in addition to clinical parameters [8,9]. According to the Assessment of SpondyloArthritis International Society/European Alliance of Associations for Rheumatology (ASAS–EULAR) 2022 recommendations, ASDAS is the preferred measure for monitoring disease activity and guiding treatment decisions, including the initiation of biologic therapy [10].

In addition to musculoskeletal manifestations, AS is associated with cardiometabolic comorbidity. A recent meta-analysis reported a pooled prevalence of metabolic syndrome of 15.5% (95% CI: 10.9–20.8%) among patients with AS [11]. Population-based data also indicate evolving epidemiology and treatment patterns in AS [12].

Brain-derived neurotrophic factor (BDNF), a member of the neurotrophins family, contributes to neuronal survival, synaptic plasticity, pain modulation, and inflammatory signaling [13,14]. BDNF has also been linked to metabolic and cardiovascular phenotypes [15]. Its expression and function are influenced by genetic variation and other regulatory mechanisms.

The *BDNF* gene is located on chromosome 11p14.1 and has a complex transcriptional organization involving alternative promoters and transcript isoforms [16,17].

The most extensively investigated genetic variant of the *BDNF* gene is the Val66Met polymorphism (rs6265; BDNF NM_170735.6:c.196G>A, p.Val66Met). This single-nucleotide polymorphism consists of a guanine-to-adenine substitution at nucleotide position 196 (G196A), resulting in a valine-to-methionine substitution at codon 66 (Val66Met). Because BDNF is transcribed from the minus strand, dbSNP reports rs6265 as C>T on the plus strand; throughout this manuscript we use the G>A notation corresponding to the minus-strand/coding convention. It has been associated with molecular, cellular, and structural brain changes, as well as alterations in cognitive and social functioning [18].

The Val66Met substitution occurs in the BDNF prodomain and affects intracellular trafficking and activity-dependent secretion of BDNF [19]. These functional effects provide a biological rationale for investigating rs6265 as a potential modifier of pain processing and other clinical phenotypes.

Because of its biological relevance, the Val66Met polymorphism has been extensively studied in both healthy individuals and patients with neuropsychiatric disorders. Reported associations have been inconsistent, likely reflecting the influence of age, sex, ethnicity, environmental exposures, and interactions with other genetic factors [16]. Marked differences in allele frequencies have been described among populations worldwide. In European populations, the Met allele frequency is generally reported between 10% and 20% [20], while a study performed in a healthy Romanian population reported frequencies of 80.74% for the Val allele and 19.26% for the Met allele [21].

Chronic pain is a major determinant of disease burden in AS. BDNF has been implicated in pain modulation and central sensitization [22], and the Val66Met polymorphism may influence pain processing and habituation [23]. In animal models, dendritic targeting of *Bdnf* mRNA is essential for energy homeostasis and leptin responsiveness [24]. In humans, the Val66Met polymorphism has been investigated in relation to obesity, insulin resistance, dyslipidemia, and type 2 diabetes mellitus [25,26,27].

The Val66Met polymorphism has also been investigated in chronic pain disorders. In fibromyalgia, genotype frequencies did not differ from controls, although metabolic and inflammatory differences were reported within clinical subgroups [28]. These observations, together with the established role of BDNF in central sensitization [22,23], support investigation of Val66Met in rheumatic pain phenotypes.

Beyond its neurological functions, BDNF participates in immune and inflammatory pathways [29,30]. Experimental studies indicate that BDNF signaling can modulate macrophage cytokine responses [31], and Val66Met has been investigated as a modifier of inflammatory and neurodegenerative processes in multiple sclerosis [32].

A recent study measured serum BDNF, proBDNF, and sortilin in 74 axSpA patients versus 79 matched healthy controls and reported reduced serum BDNF in axSpA, sex-related variability, a lower BDNF/proBDNF ratio in patients with depression, and an inverse relationship between CRP and BDNF levels [33]; a companion study examined inflammation and serum adipocytokines in relation to depression and anxiety burden in axSpA [34]. These findings are directly relevant to the present rationale, and the reported inverse relationship between inflammation and circulating BDNF offers a plausible explanation for why a genotype effect could be attenuated in an inflamed cohort such as ours (see Section 4.3). To evaluate the novelty of this question, PubMed and Scopus were searched on 2 September 2026 using combinations of (“BDNF” OR “brain-derived neurotrophic factor” OR “Val66Met” OR “rs6265”) AND (“ankylosing spondylitis” OR “axial spondyloarthritis” OR “radiographic axial spondyloarthritis”). No previous study evaluating rs6265 genotype in relation to disease activity in AS or axial spondyloarthritis was identified.

Given the involvement of BDNF in pain processing, inflammatory signaling, depressive symptoms, and metabolic regulation, the Val66Met polymorphism is a plausible modifier of the AS phenotype. This study evaluated the distribution of the BDNF Val66Met (rs6265) genotype and explored its associations with disease activity, inflammatory burden, pain, depressive symptoms, and cardiometabolic characteristics in patients with AS. ASDAS-CRP was designated as the primary clinical outcome. BASDAI, pain NRS, HADS-D score, and metabolic parameters were treated as secondary or exploratory outcomes.

## 2. Materials and Methods

### 2.1. Study Design and Patient Population

This cross-sectional study included 81 patients diagnosed with AS who were recruited from the Rheumatology Department of Bihor County Emergency Clinical Hospital, Bihor, Romania, between 1 March and 30 June 2026. All patients fulfilled the modified New York criteria for AS [35]. Under current ASAS nomenclature, these patients correspond to radiographic axial spondyloarthritis (r-axSpA); the term AS is retained throughout the manuscript for readability and consistency with the cited literature. Patients were recruited consecutively, with all eligible patients presenting during the study period included without additional selection. HLA-B27 positivity was not an eligibility criterion. All 81 consecutively recruited participants had previously undergone laboratory testing for HLA-B27 and had a documented positive result before enrolment. HLA-B27 status was obtained from laboratory documentation and was not inferred from the clinical diagnosis. The specific testing method and performing laboratory could not be uniformly retrieved from the available medical records.

DNA samples from all 81 participants were sent for BDNF genotyping at the Department of Cell and Molecular Biology, Iuliu Hațieganu University of Medicine and Pharmacy, Cluj-Napoca, Romania. All 81 samples yielded a valid genotype; four samples required repeat amplification because of suboptimal agarose-gel electrophoretic profiles on the first attempt. There was no attrition between recruitment and the final genotype analysis. The cohort comprised 61 men and 20 women, with a mean age of 53.2 ± 12.5 years (range, 21–77 years).

Current treatment information was available for 80 participants. Twenty-seven patients were receiving biologic therapy with a TNF-α or IL-17 inhibitor, and 53 were receiving non-biologic treatment. One participant had missing treatment information and was excluded from treatment-stratified comparisons and adjusted regression models. Among the 53 participants receiving non-biologic treatment, 37 were treated with nonsteroidal anti-inflammatory drugs, 11 with sulfasalazine, four with methotrexate, and one with combined sulfasalazine and methotrexate. Current biologic-therapy status did not necessarily indicate biologic monotherapy, because concomitant conventional treatment could also be used.

### 2.2. Clinical and Biochemical Data Collection

For each participant, the latest recorded demographic, anthropometric, clinical, and laboratory data were collected from medical records. Data were abstracted retrospectively, and for each variable the most recently recorded value prior to genotyping was used; a strict maximum interval between clinical assessment, questionnaires, and laboratory testing was not enforced. We acknowledge this as a limitation: correlations between variables that may have been recorded at different time points (e.g., BASDAI and CRP) should be interpreted with appropriate caution. Anthropometric measurements included body weight, height, waist circumference, and body mass index (BMI). Waist circumference was measured following the standard WHO protocol: with the patient standing, at the midpoint between the lower costal margin and the iliac crest, using a non-stretch tape, at the end of a normal expiration [36]. Blood pressure was measured with the patient seated and at rest for at least 5 min using a standard sphygmomanometer on the non-dominant arm; the recorded value corresponded to the reading obtained during the clinical visit closest to the other assessments. Antihypertensive, lipid-lowering, and glucose-lowering medication use was not systematically recorded in this dataset and could not be quantified; because standard metabolic syndrome criteria count a treated patient as meeting the relevant component regardless of the achieved value, this omission likely results in an underestimation, rather than an overestimation, of the true metabolic syndrome prevalence in this cohort. Lifestyle variables included smoking status, alcohol consumption, and level of physical activity. Laboratory parameters comprised serum triglycerides, HDL-cholesterol, LDL-cholesterol, fasting glycemia, and C-reactive protein (CRP). Disease activity was assessed using the Bath Ankylosing Spondylitis Disease Activity Index (BASDAI) [7] and the Ankylosing Spondylitis Disease Activity Score using CRP (ASDAS-CRP) [8], pain intensity using a Numerical Rating Scale (NRS, 0–10), and depressive symptoms using the depression subscale of the Hospital Anxiety and Depression Scale (HADS-D, 0–21) [37]. HADS-D scores ≥ 8 were considered indicative of possible clinically relevant depressive symptoms; HADS-D data were available for 79 of 81 patients. Patient global assessment (PGA, 0–10 scale) was collected for each patient as an additional disease-activity input. ASDAS-CRP was calculated according to the validated formula: ASDAS-CRP = 0.121 × total back pain + 0.110 × PGA + 0.073 × peripheral pain/swelling + 0.058 × duration of morning stiffness + 0.579 × ln(CRP[mg/L] + 1) [8]. Disease-activity states were categorized, per Machado et al. [9], as inactive disease (<1.3), low disease activity (≥1.3 to <2.1), high disease activity (≥2.1 to ≤3.5), or very high disease activity (>3.5). For this analysis, metabolic syndrome was operationally defined as the presence of at least three of five measured components using harmonized cut-offs: waist circumference ≥94 cm in men or ≥80 cm in women; triglycerides ≥150 mg/dL; HDL-cholesterol <40 mg/dL in men or <50 mg/dL in women; systolic blood pressure ≥130 mmHg and/or diastolic blood pressure ≥85 mmHg; and fasting glycemia ≥100 mg/dL [38,39]. Medication use for these components was not incorporated into classification because medication data were not systematically available.

### 2.3. Genotyping of the BDNF Val66Met Polymorphism

Genomic DNA was extracted from peripheral whole blood collected in EDTA tubes using the QuickGene automated nucleic acid isolation system and the QuickGene DNA Whole Blood Kit S (Kurabo Industries Ltd., Osaka, Japan), according to the manufacturer’s instructions. DNA extracts were stored at −80 °C until PCR analysis.

The *BDNF* Val66Met polymorphism (rs6265; c.196G>A) was genotyped using tetra-primer amplification refractory mutation system PCR (T-ARMS-PCR) based on the general method described by Ye et al. [40] and the assay developed for BDNF rs6265 by Wang et al. [41]. Each 20 µL reaction contained 2 µL of genomic DNA, 4 µL of 5× FIREPol Ready to Load Master Mix containing 7.5 mM MgCl_2_ (Solis BioDyne, Tartu, Estonia), 12 µL of nuclease-free water, 0.25 µL of each outer primer (OF and OR; 10 µM stock concentration), and 0.75 µL of each inner allele-specific primer (IF and IR; 10 µM stock concentration). The final MgCl_2_ concentration was 1.5 mM. Primer sequences and final concentrations are provided in Supplementary Table S1.

Amplification was performed using a Bio-Rad CFX96 thermal cycler (Bio-Rad Laboratories) under the following touchdown conditions: initial denaturation at 94 °C for 3 min; 30 cycles of denaturation at 94 °C for 30 s, annealing for 30 s, and extension at 72 °C for 30 s; followed by a final extension at 72 °C for 5 min. The annealing temperature was decreased from 63 °C to 57 °C by 0.3 °C per cycle during the first 20 cycles and was maintained at 57 °C for the remaining 10 cycles. The cycling protocol is illustrated in Supplementary Figure S1.

PCR products were separated by electrophoresis on 3% agarose gels, stained with Midori Green (NIPPON Genetics Europe GmbH, Düren, Germany), and visualized under ultraviolet light. The common outer-primer amplicon was 406 bp, while the G (Val)- and A (Met)-specific products were 253 bp and 203 bp, respectively. Accordingly, GG genotypes were identified by bands of 406 and 253 bp; GA genotypes by bands of 406, 253, and 203 bp; and AA genotypes would have been identified by bands of 406 and 203 bp. The primer configuration and expected products are illustrated in Supplementary Figure S2, and representative electrophoretic profiles are shown in Supplementary Figures S3 and S4.

Genotype calls were independently assigned by three investigators who interpreted the gels without access to the participants’ individual clinical or biochemical data. Samples showing suboptimal or ambiguous electrophoretic profiles were re-amplified before a final genotype was assigned. To assess the reproducibility of the genotyping results, 10 samples were randomly selected and reanalyzed in a blinded manner; six were heterozygous (GA) and four were homozygous (GG), with complete concordance with the initial genotyping results. No-template controls and positive controls with known genotypes were not included consistently in every experimental run. Genotype calls were not independently confirmed by Sanger sequencing or PCR-RFLP.

### 2.4. Statistical Analysis

Statistical analyses were performed using Python version 3.12.14 with SciPy version 1.17.0, NumPy version 2.3.5, and pandas version 2.2.3, together with R software version 4.3.1. Microsoft Excel version 16.108.1 was used for data entry, source-data verification, and database management, but not for inferential statistical analyses.Data distribution was assessed using the Shapiro–Wilk test and visual inspection of histograms and Q–Q plots. Quantitative variables were presented as mean ± standard deviation (SD) when approximately normally distributed and as median and interquartile range (IQR) otherwise. Between-group comparisons were performed using Welch’s independent-samples *t*-test or the Mann–Whitney U test, as appropriate. For *t*-test comparisons, mean differences with 95% confidence intervals (CIs) and the absolute value of Cohen’s d were reported. For Mann–Whitney U comparisons, Hodges–Lehmann location differences with 95% CIs estimated using a nonparametric percentile bootstrap with 50,000 resamples and the absolute value of the rank-biserial correlation were reported. Categorical variables were compared using the chi-square test or Fisher’s exact test, as appropriate. Associations between continuous variables were assessed using Spearman’s rank correlation coefficient. To account for multiple testing, *p*-values from the 28 pairwise Spearman correlations were adjusted using the Benjamini–Hochberg false discovery rate procedure; q < 0.05 was considered statistically significant after correction. Hardy–Weinberg equilibrium was evaluated using an exact test [42]. All tests were two-sided. For analyses other than the FDR-adjusted correlation analysis, *p* < 0.05 was considered statistically significant.

To examine whether BDNF genotype was associated with disease activity independently of potential confounders, exploratory multivariable linear regression models were fitted for ASDAS-CRP, the primary clinical outcome, and BASDAI, a secondary outcome. Both models included genotype (GA versus GG), age, sex, disease duration, current biologic-therapy status, and current smoking status as covariates. An exploratory multivariable binary logistic regression model was also fitted for metabolic syndrome using the same covariates. Unstandardized regression coefficients with 95% confidence intervals (CIs) were reported for the linear models, and odds ratios with 95% CIs were reported for the logistic model. Given the limited sample size and the number of covariates, all adjusted analyses were interpreted as exploratory. The Benjamini–Hochberg false discovery rate procedure was applied across the 28 pairwise Spearman correlation tests; multiplicity was not formally controlled for the remaining secondary and exploratory analyses.

All values were checked against physiologically plausible ranges, including age 21–77 years, CRP 1.7–48.6 mg/L, BMI 18.2–33.7 kg/m^2^, waist circumference 74–107 cm, systolic blood pressure 110–175 mmHg, and diastolic blood pressure 67–95 mmHg. Range screening did not identify physiologically implausible values; however, six waist-circumference entries were discrepant with the original medical records despite remaining within plausible adult ranges. Using anonymous correction identifiers, the previous and source-verified values were: C1, GA, 90/103 cm; C2, GG, 79/105 cm; C3, GG, 87/102 cm; C4, GA, 89/98 cm; C5, GA, 90/107 cm; and C6, GA, 87/98 cm. All six participants were men, and each source-verified value crossed the harmonized male waist-circumference threshold of 94 cm. Initial anthropometric measurements were performed by the first author before BDNF genotyping results became available. Retrospective verification of the six records was performed by the first author after genotype results had been entered into the study database and was not blinded to genotype. The database was corrected according to the original medical documentation, the metabolic-syndrome classification and all analyses involving waist circumference were recalculated, and no participant was excluded from the primary analysis. The study was reported in accordance with the STREGA extension of the STROBE statement [43]. A completed STREGA checklist is provided as Supplementary Table S3.

## 3. Results

### 3.1. Demographic, Therapeutic and Lifestyle Characteristics of the Cohort

The study cohort included 81 patients with AS, comprising 61 men (75.3%) and 20 women (24.7%). The mean age was 53.2 ± 12.5 years (range, 21–77 years), while the median age at diagnosis was 29 years (IQR, 23–37 years). Forty-six participants (56.8%) were diagnosed before the age of 30 years, and four (4.9%) were diagnosed before the age of 16 years. The median disease duration was 23 years (IQR, 14–29 years). Of the 81 participants, 27 (33.3%) were receiving biologic therapy, 53 (65.4%) were receiving non-biologic treatment, and one (1.2%) had missing current-treatment information. Among the 53 participants receiving non-biologic treatment, 37 received nonsteroidal anti-inflammatory drugs, 11 sulfasalazine, four methotrexate, and one combined sulfasalazine and methotrexate. Waist circumference was 88.3 ± 7.1 cm in men and 83.5 ± 7.4 cm in women. Within-sex analyses also showed no significant BASDAI difference by genotype. Among men, the mean BASDAI was 3.6 ± 1.3 in GG carriers and 3.5 ± 1.4 in GA carriers (Welch’s *t*-test *p* = 0.872). Among women, the mean BASDAI was 3.4 ± 1.8 in GG carriers and 4.1 ± 1.4 in GA carriers (Welch’s *t*-test *p* = 0.351).

Demographic, therapeutic, and lifestyle characteristics according to *BDNF* Val66Met genotype are presented in Table 1.

No significant differences were observed between the GG (Val/Val) and GA (Val/Met) genotype groups regarding sex, age, age-at-diagnosis category, disease duration, or current biologic-therapy status (all *p* > 0.05). Current smoking was reported by 13 of 57 participants with the GG genotype and by none of the 24 participants with the GA genotype. The overall three-category smoking distribution differed between genotype groups in the unadjusted analysis (Fisher–Freeman–Halton *p* = 0.025). However, this exploratory observation is fragile because the GA group contained a zero cell, and current smoking was not statistically significant in the adjusted multivariable models. Therefore, the unadjusted finding should not be interpreted as evidence of an independent association between genotype and smoking status. Disease activity did not differ significantly by sex: the median BASDAI was 3.5 (IQR, 2.4–4.5) in men and 3.4 (IQR, 2.6–4.9) in women (*p* = 0.730), while the median ASDAS-CRP was 2.53 (IQR, 1.96–3.06) in men and 2.66 (IQR, 2.20–3.06) in women (*p* = 0.697).

### 3.2. BDNF Val66Met Genotype Distribution and Allele Frequencies

Of the 81 samples analyzed, 77 yielded clear and interpretable genotype profiles on the first analysis, corresponding to a first pass call rate of 95.1%. Four samples with suboptimal electrophoretic profiles were re-amplified and subsequently yielded interpretable results, resulting in a final call rate of 81/81 (100%). No samples were excluded because of genotyping failure. To assess the reproducibility of the genotyping results, 10 samples were randomly selected and reanalyzed in a blinded manner; six were heterozygous (GA) and four were homozygous (GG), with complete concordance with the initial genotyping results.

T-ARMS-PCR assigned 57 participants (70.4%) to the GG (Val/Val) genotype and 24 (29.6%) to the GA (Val/Met) genotype; no AA (Met/Met) banding pattern was observed. The G (Val) allele frequency was 85.2%, and the A (Met) allele frequency was 14.8%. Because the genotype calls were not independently confirmed by Sanger sequencing or PCR-RFLP, the absence of AA homozygotes should be regarded as an unconfirmed observation.

**Figure 1:**
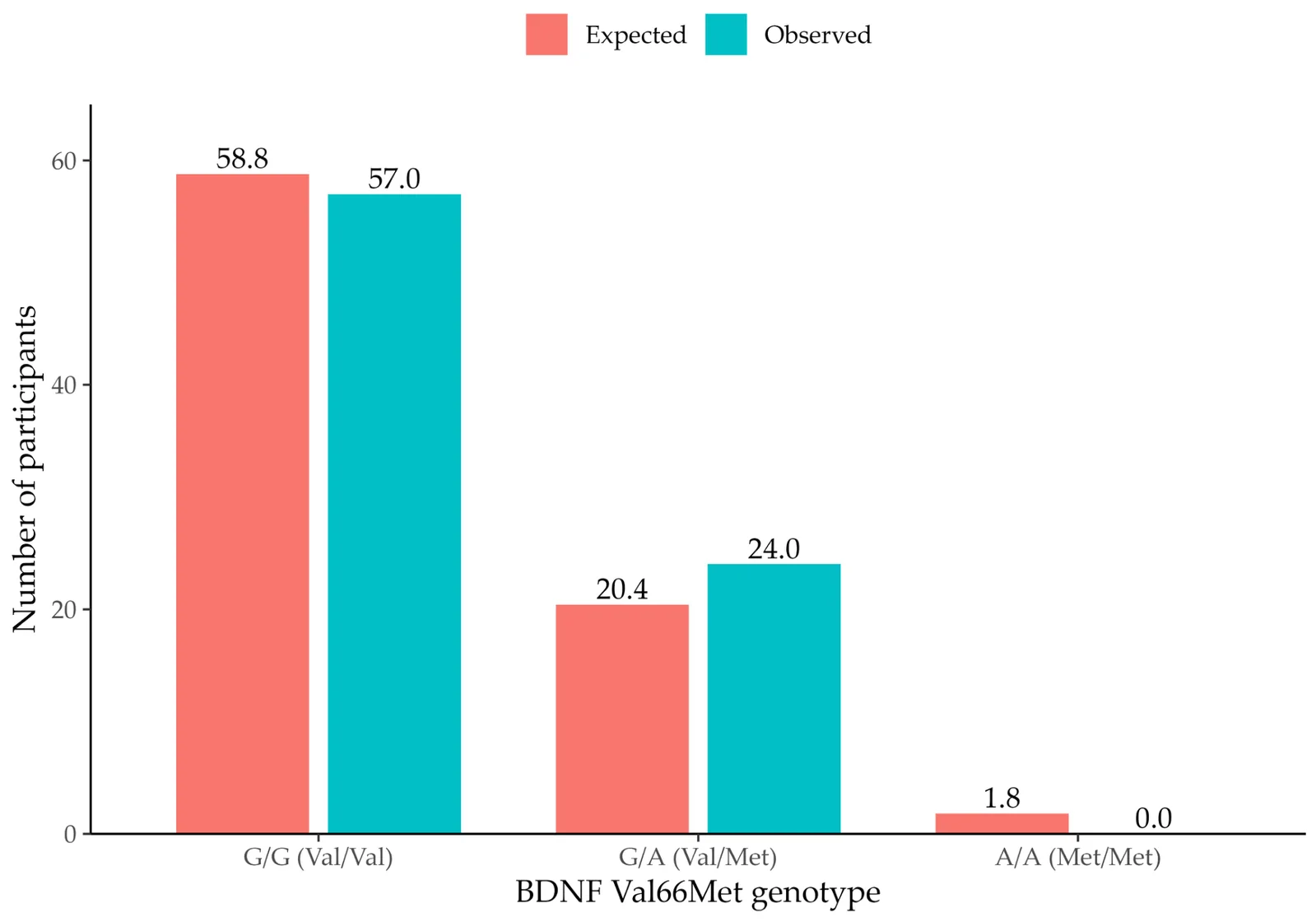
Observed and Hardy–Weinberg equilibrium-expected counts for the BDNF Val66Met genotypes. The small expected number of AA homozygotes limits the ability of the HWE test to detect potential genotype-calling errors affecting this class.

The observed genotype counts were GG = 57, GA = 24, and AA = 0, compared with HWE-expected counts of 58.8, 20.4, and 1.8, respectively (Figure 1). The genotype distribution did not show a statistically significant deviation from Hardy–Weinberg equilibrium (exact *p* = 0.198) [42]. However, given the small expected number of AA homozygotes, the HWE test had limited ability to detect genotype-calling errors affecting the rare homozygous class and was not interpreted as evidence of genotyping accuracy.

### 3.3. Clinical and Metabolic Characteristics of the Cohort

Clinical therapeutic and lifestyle characteristics were compared between carriers of the GG (Val/Val) and GA (Val/Met) genotypes (Table 2).

Observed ranges (min-max) for the Table 2 variables were: BASDAI 0.4–6.9; CRP 1.7–48.6 mg/L; pain NRS 0.0–8.0; HADS-D 2.0–17.0; BMI 18.2–33.7 kg/m2; waist circumference 74.0–107.0 cm; triglycerides 94.0–208.0 mg/dL; HDL-C 27.0–74.0 mg/dL; LDL-C 68.0–199.0 mg/dL; fasting glucose 64.0–185.0 mg/dL; systolic BP 110.0–175.0 mmHg; and diastolic BP 67.0–95.0 mmHg. ASDAS-CRP, the primary clinical outcome, was also similar between the genotype groups, with mean values of 2.56 ± 0.66 in the GG group and 2.49 ± 0.72 in the GA group (*p* = 0.717; effect size = 0.10).

**Figure 2:**
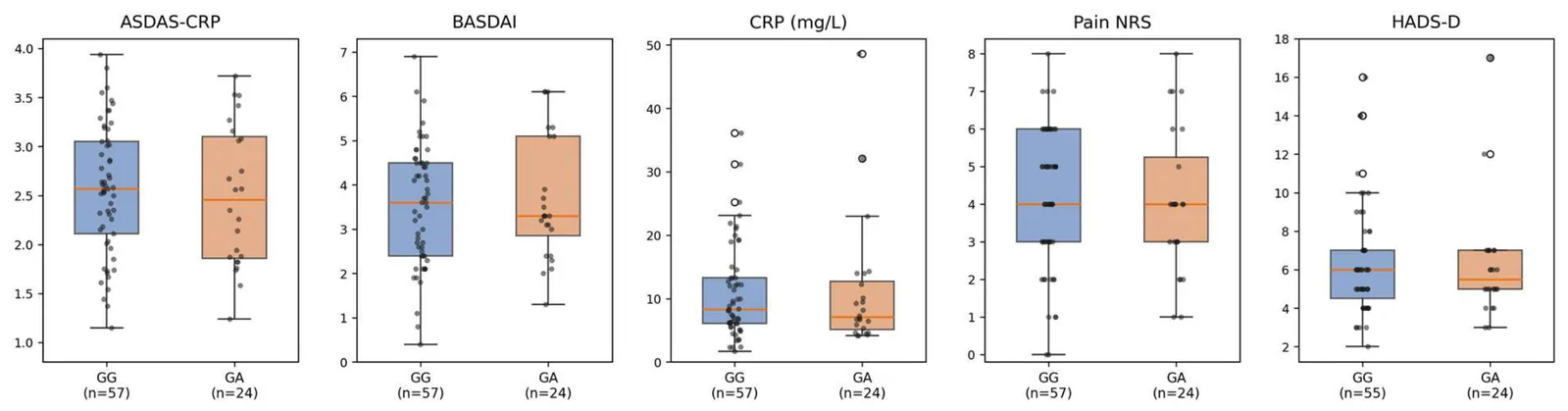
ASDAS-CRP, BASDAI, CRP, pain NRS, and HADS-D by BDNF genotype, with individual data points.

Disease activity assessed using BASDAI was similar between the two genotype groups (3.5 ± 1.4 in GG carriers vs. 3.7 ± 1.4 in GA carriers; *p* = 0.668) (Figure 2). Likewise, CRP levels did not differ significantly between groups (8.3 (6.1–13.3) vs. 7.1 (5.1–12.7) mg/L; *p* = 0.721). Pain intensity measured by NRS was comparable (4 (3–6)vs. 4 (3–5.2); *p* = 0.579), as were HADS-D scores (6 (4.5–7)vs. 5.5 (5–7); *p* = 0.829). Extra-articular manifestations included uveitis in 6 patients (7.4%), psoriasis in 5 (6.2%), and inflammatory bowel disease in 3 (3.7%). No statistically significant differences were observed in these manifestations. None of the metabolic parameters analyzed (triglycerides, HDL-C, LDL-C, blood glucose, BMI, waist circumference) showed a statistically significant association with the genotype studied (all *p* > 0.05). Effect sizes were generally small [44], with no statistically significant between-genotype differences observed. No statistically significant differences were observed in systolic or diastolic blood pressure between the two genotype groups (*p* = 0.860 and *p* = 0.143, respectively).

### 3.4. Cardiometabolic Profile in the Study Cohort

Cardiometabolic risk factors were frequent in the study cohort (Table 3). Elevated triglycerides (≥150 mg/dL) were present in 24 participants (29.6%), low HDL-C in 32 (39.5%), elevated blood pressure (systolic BP ≥130 mmHg and/or diastolic BP ≥85 mmHg) in 61 (75.3%), fasting glycemia ≥100 mg/dL in 18 (22.2%), and elevated waist circumference (≥94 cm in men or ≥80 cm in women) in 27 (33.3%). The distribution of serum triglyceride concentrations is shown in Supplementary Figure S5.

Using the harmonized Joint Interim Statement definition applied throughout the study, 24 participants (29.6%) met at least three of the five measured criteria for metabolic syndrome. Medication use for the individual metabolic components was not incorporated because these data were not systematically available. As a sensitivity analysis, applying the NCEP-ATP III waist-circumference thresholds (≥102 cm in men and ≥88 cm in women), while leaving the other four components unchanged, resulted in 18 participants (22.2%; 13 men and 5 women) meeting the criteria for metabolic syndrome. Under these alternative thresholds, the elevated waist-circumference criterion was met by 4 of 61 men and 6 of 20 women. The retrospective source-data verification of the six corrected waist-circumference records was performed with knowledge of the participants’ genotypes and was therefore not blinded.

In a separate sensitivity analysis excluding the six participants with corrected waist-circumference entries (n = 75; GA n = 20; GG n = 55), the elevated-waist criterion was met by 8/20 GA carriers (40.0%) and 13/55 GG carriers (23.6%; exact conditional OR = 2.13, 95% CI 0.61–7.23; Fisher’s exact *p* = 0.244). Metabolic syndrome was present in 6/20 GA carriers (30.0%) and 12/55 GG carriers (21.8%; exact conditional OR = 1.53, 95% CI 0.39–5.47; Fisher’s exact *p* = 0.544). Neither association was statistically significant. BMI and waist circumference showed a moderate positive Pearson correlation (r = 0.519, *p* < 0.001); in a simple linear model, waist circumference increased by 1.08 cm per 1 kg/m^2^ increase in BMI.

No statistically significant association was observed between BDNF Val66Met genotype and metabolic syndrome in the unadjusted analysis (GA versus GG: OR = 2.17, 95% CI 0.70–6.73, Fisher’s exact *p* = 0.182). The adjusted exploratory logistic regression model is reported in Section 3.5.

In a sensitivity analysis excluding the six participants whose waist-circumference values were corrected, 75 participants remained (55 GG and 20 GA). The harmonized waist-circumference criterion was met by 8 of 20 GA carriers (40.0%) and 13 of 55 GG carriers (23.6%; exact conditional OR = 2.13, 95% CI 0.61–7.23; Fisher’s exact *p* = 0.244). Metabolic syndrome was present in 6 of 20 GA carriers (30.0%) and 12 of 55 GG carriers (21.8%; exact conditional OR = 1.53, 95% CI 0.39–5.47; Fisher’s exact *p* = 0.544). The direction of both estimates was unchanged, but the associations were attenuated and remained statistically non-significant.

### 3.5. Exploratory Analyses by Biologic Therapy Status and Adjusted Regression Models

In a secondary exploratory analysis, 27 patients were receiving current biologic therapy and 53 were receiving non-biologic treatment. One participant had missing treatment information and was excluded from the treatment-stratified comparisons and adjusted regression models; therefore, these analyses included 80 participants. Biologic therapy refers to current treatment status and does not necessarily indicate biologic monotherapy (Table 4).

Patients receiving biologic therapy had significantly lower CRP, ASDAS-CRP, BASDAI, pain NRS, and HADS-D values than patients receiving non-biologic treatment (*p* < 0.05 for all comparisons). Age also differed between the groups, with a median age of 51.0 years (IQR, 41.0–56.0) in patients receiving biologic therapy and 58.0 years (IQR, 48.0–66.0) in those receiving non-biologic treatment (*p* = 0.005). No statistically significant between-group differences were observed for disease duration, triglycerides, HDL-C, LDL-C, fasting glycemia, BMI, or waist circumference.

By ASDAS-CRP, the full cohort showed substantial disease activity, with a mean score of 2.5 ± 0.7 and 71.6% of participants classified as having high or very high disease activity. This contrasted with the mean BASDAI score of 3.6, which was below the conventional high-disease-activity threshold of ≥4; 31 of 81 participants (38.3%) had BASDAI ≥ 4. This difference reflects the construction of the two indices: BASDAI is entirely patient-reported, whereas ASDAS-CRP also incorporates an objective inflammatory marker.

In the multivariable linear regression model for ASDAS-CRP (n = 80), BDNF genotype was not associated with the outcome after adjustment for age, sex, disease duration, current biologic-therapy status, and current smoking status (B = 0.045, 95% CI −0.238 to 0.328, *p* = 0.754). Current biologic therapy was associated with a lower ASDAS-CRP score (B = −0.942, 95% CI −1.222 to −0.662, *p* < 0.001), whereas current smoking status was not significantly associated with ASDAS-CRP (B = 0.209, 95% CI −0.171 to 0.589, *p* = 0.276). The model explained 42.4% of the variability in ASDAS-CRP (R^2^ = 0.424; adjusted R^2^ = 0.377; F(6,73) = 8.96, *p* < 0.001).

In the secondary multivariable linear regression model for BASDAI (n = 80), BDNF genotype was not associated with the outcome after adjustment for the same covariates (B = 0.175, 95% CI −0.454 to 0.804, *p* = 0.581). Current biologic therapy was associated with a lower BASDAI score (B = −1.425, 95% CI −2.048 to −0.802, *p* < 0.001), whereas current smoking status was not significantly associated with BASDAI (B = 0.228, 95% CI −0.615 to 1.071, *p* = 0.591). The model explained 30.4% of the variability in BASDAI (R^2^ = 0.304; adjusted R^2^ = 0.246; F(6,73) = 5.31, *p* < 0.001).

In the exploratory multivariable logistic regression model for metabolic syndrome (n = 80), BDNF genotype was not associated with the outcome after adjustment for age, sex, disease duration, current biologic-therapy status, and current smoking status (OR = 1.31, 95% CI 0.40–4.30, *p* = 0.658). Current smoking status was also not significantly associated with metabolic syndrome (OR = 0.60, 95% CI 0.11–3.44, *p* = 0.570). Because 24 participants met the criteria for metabolic syndrome and the model included six predictors, the estimates are imprecise and should be interpreted as exploratory.

Current smoking was observed in 13 of 57 participants with the GG genotype and in none of the 24 participants with the GA genotype. This association should be interpreted cautiously because it is based on a zero cell in the GA group.

### 3.6. Correlation Analyses

Spearman rank correlation analyses were performed for all 28 pairwise combinations of pain NRS, BASDAI, HADS-D, CRP, age, age at diagnosis, systolic blood pressure, and BMI. After application of the Benjamini–Hochberg false discovery rate procedure across the 28 tests, six correlations remained statistically significant at q < 0.05 (Table 5).

Pain NRS correlated positively with BASDAI (r = 0.754, q < 0.0001), HADS-D (r = 0.378, q = 0.0030), and CRP (r = 0.372, q = 0.0030). BASDAI correlated positively with HADS-D (r = 0.536, q < 0.0001), and systolic blood pressure correlated positively with BMI (r = 0.588, q < 0.0001). Age correlated with age at diagnosis (r = 0.467, q < 0.0001); however, this association was structurally expected and was not interpreted as an independent clinical finding. Complete results for all 28 pairwise correlations are provided in Supplementary Table S2.

## 4. Discussion

### 4.1. BDNF Val66Met Genotype Distribution in Ankylosing Spondylitis

The genotype distribution observed in the present cohort was 70.4% GG, 29.6% GA, and 0% AA, with a Met allele frequency of 14.8%. The Met allele frequency in the present cohort was 14.8%. Comparisons with the ALFA European population and the Romanian cohort reported by Vulturar et al. showed no statistically significant differences in allele frequencies (*p* ≈ 0.16 and *p* ≈ 0.15, respectively) [21]. These comparisons are therefore descriptive only. Because the study did not include a matched healthy control group, these external comparisons are descriptive and should not be interpreted as evidence of disease susceptibility.

Conversely, an AA homozygous sample previously analyzed by Chiș et al. using the same rs6265 T-ARMS-PCR assay, including the same primers, master mix, and cycling protocol, produced the expected A-specific banding pattern (Supplementary Figure S4), confirming that the A-specific primer was functional under these assay conditions [45]. Nevertheless, undetected genotype misclassification cannot be completely excluded, particularly for the rare AA homozygous class [46].

The genotype distribution did not show a statistically significant deviation from Hardy–Weinberg equilibrium (exact *p* = 0.198) [42]. However, because only 1.8 AA homozygotes were expected under HWE, this test had limited ability to detect genotype-calling errors affecting the rare homozygous class and was not interpreted as evidence of genotyping accuracy. Based on the AA genotype frequency of 3.7% reported for the ALFA European population, the probability of observing no AA homozygotes in a sample of 81 individuals is approximately 4.7%. Based on the AA frequencies of 4.00% and 5.26% reported in two previous Romanian cohorts, the corresponding probabilities are approximately 3.7% and 1.3%, respectively. Thus, the absence of an AA banding pattern represents a borderline or uncommon finding. It may have occurred by chance, but it is also compatible with, although not proof of, undetected genotype misclassification. In the absence of confirmation by an independent genotyping method, the observation of no AA homozygotes remains unconfirmed.

### 4.2. Absence of Genotype–Phenotype Associations

No statistically significant differences were observed between GG and GA carriers for the clinical, inflammatory, or metabolic variables evaluated. In the adjusted model, BDNF genotype was not associated with ASDAS-CRP after adjustment for age, sex, disease duration, current biologic-therapy status, and current smoking status (B = 0.045, 95% CI −0.238 to 0.328, *p* = 0.754). Similarly, genotype was not associated with BASDAI in the adjusted secondary model (B = 0.175, 95% CI −0.454 to 0.804, *p* = 0.581). More broadly, Val66Met association studies have produced heterogeneous and frequently non-replicated findings, partly because of limited statistical power, population differences, and variability in the phenotypes examined [16]. These findings should be interpreted as exploratory rather than as evidence of absence of a smaller genotype-associated effect.

Interpretation of these negative findings is limited by the modest sample size, including only 24 GA heterozygotes and no confirmed AA homozygotes. The study had 80% power to detect only relatively large between-genotype differences (approximately Cohen’s d ≥ 0.69), and smaller associations cannot be excluded. The absence of a healthy control group also precluded direct assessment of whether genotype or allele frequencies differed between patients with AS and the general population.

The Benjamini–Hochberg procedure was applied across the 28 pairwise correlation tests, and six correlations remained significant at q < 0.05. Multiplicity was not formally controlled for the remaining secondary and exploratory analyses. Consequently, all findings should be interpreted as hypothesis-generating rather than confirmatory. Any effect of the Val66Met polymorphism in systemic inflammatory disease may be modest and potentially obscured by genetic, environmental, disease-related, and treatment-related heterogeneity.

The HADS-D analysis showed that 19.0% of participants (15 of 79 with available scores) had HADS-D scores ≥8, indicating possible clinically relevant depressive symptoms. However, HADS-D scores did not differ according to genotype. This finding is consistent with previous studies reporting inconsistent associations between the Val66Met polymorphism and depression- or anxiety-related phenotypes across different populations [16,47].

### 4.3. Disease Activity, Pain, and Depressive Symptoms

The strong correlation observed between pain intensity (NRS) and BASDAI score (r = 0.754, *p* < 0.0001, q < 0.0001) is consistent with the central role of pain in patient-reported disease activity, while also reflecting partial construct overlap because BASDAI includes pain-related items [9,10]. Significant correlations were also observed between BASDAI and HADS-D score (r = 0.536, *p* < 0.0001, q < 0.0001) and between pain NRS and HADS-D score (r = 0.378, *p* = 0.0006, q = 0.0030), supporting a close relationship between disease activity, pain perception, and depressive symptom burden in patients with AS [48,49].

A meta-analysis reported a pooled prevalence of depression of approximately 35% among patients with AS and demonstrated higher disease activity scores in patients with depressive symptoms than in those without depression [47]. Our findings are consistent with these observations and emphasize the multidimensional nature of disease burden in AS.

The significant correlation between pain NRS and CRP (r = 0.372, *p* = 0.0006, q = 0.0030) suggests that pain intensity in this cohort was related, at least in part, to ongoing inflammatory activity. Together, the correlations that remained significant after Benjamini–Hochberg correction indicate that both inflammatory processes and depressive symptoms may contribute to symptom severity and perceived disease activity in AS.

### 4.4. Biologic Therapy Status and Disease Activity

Patients receiving biologic therapy had significantly lower BASDAI scores (2.6 ± 1.1 vs. 4.1 ± 1.2, *p* < 0.0001), ASDAS-CRP scores (1.9 ± 0.4 vs. 2.8 ± 0.6, *p* < 0.0001), and CRP levels (median [IQR]: 5.9 [4.4–7.4] vs. 12.0 [6.7–15.0] mg/L, *p* < 0.0001) than patients receiving non-biologic treatment. Because ASDAS-CRP incorporates CRP directly in its formula, the ASDAS-CRP difference between treatment groups is not independent of the CRP difference reported in the same comparison; both are downstream of treatment allocation rather than independent lines of evidence. These between-group differences are compatible with the established efficacy of TNF-α inhibitors and IL-17 inhibitors in reducing disease activity and inflammation in AS [50,51], but the cross-sectional design does not permit estimation of a causal treatment effect.

Patients treated with biologic agents were significantly younger than those receiving non-biologic therapy (median 51.0 years [IQR, 41.0–56.0] vs. 58.0 years [IQR, 48.0–66.0], *p* = 0.005). This difference may reflect treatment patterns, referral practices, or prescribing preferences within the studied population.

Among the selected metabolic measures included in the treatment-status comparison, triglycerides and glycemia did not differ significantly between biologic and non-biologic groups. Previous reports suggest that the metabolic effects of biologic therapies are variable and often modest [52,53]. The present cross-sectional study was not designed to evaluate treatment-related metabolic effects directly [54].

### 4.5. Cardiometabolic Profile and Cardiovascular Risk in the Cohort

Metabolic syndrome was present in 29.6% (24/81) of patients in our cohort using the threshold-based criteria applied in this study [38]. This proportion is nearly twice the pooled estimate of 15.5% reported in a recent meta-analysis of patients with AS [11], but lower than the general-population prevalence of 38.5% reported for the adult Romanian population in the PREDATORR survey [55]. Direct comparisons across studies should nevertheless be interpreted cautiously because prevalence estimates are influenced by age and sex distribution, disease characteristics, the criteria used to define metabolic syndrome, and whether medication use is incorporated into component classification.

Among individual metabolic abnormalities, elevated blood pressure was the most frequent component, affecting 75.3% of patients, followed by low HDL-cholesterol (39.5%), elevated waist circumference (33.3%), hypertriglyceridemia (29.6%), and fasting glycemia ≥100 mg/dL (22.2%). Of the 61 patients meeting the elevated BP criterion, 41 (67.2%) met it by systolic BP alone, 4 (6.6%) by diastolic BP alone, and 16 (26.2%) by both. Because metabolic syndrome requires at least three of five components, the high prevalence of any single component, such as elevated BP, does not translate directly into a comparably high overall metabolic syndrome prevalence; this explains the apparent tension between 75.3% elevated BP and 29.6% metabolic syndrome. These findings are consistent with previous reports demonstrating a clinically relevant burden of cardiometabolic risk factors among patients with AS [12,56]. Chronic systemic inflammation has been implicated in the development of metabolic disturbances and accelerated cardiovascular risk in patients with inflammatory rheumatic diseases, including AS [57].

A non-significant inverse correlation was observed between physical activity level and CRP concentration (r = −0.207, *p* = 0.064); this finding was considered inconclusive. Nevertheless, 69.1% of patients reported a predominantly sedentary lifestyle. Previous studies and meta-analyses have shown that exercise interventions can improve disease activity, physical function, and quality of life in patients with AS [58,59], and regular physical activity remains an important component of comprehensive disease management.

Metabolic syndrome was present in 14 of 57 GG carriers (24.6%) and 10 of 24 GA carriers (41.7%). Although this difference was not statistically significant (OR = 2.17, 95% CI 0.70–6.73; Fisher’s exact *p* = 0.182), the wide confidence interval means that the present data can neither confirm nor exclude a genotype-associated effect on metabolic risk. Likewise, none of the individual metabolic parameters evaluated differed significantly according to genotype. Although previous studies have suggested associations between the Val66Met polymorphism and obesity, insulin resistance, dyslipidemia, and type 2 diabetes mellitus [25,26,27], the present findings remain inconclusive. Given the limited sample size, the absence of AA homozygotes, and the fact that the study was powered to detect only medium-to-large effects (Cohen’s d ≥ 0.69), smaller genotype-related effects cannot be excluded.

### 4.6. Limitations

The main methodological limitation concerns genotype validation. Genotyping was performed using a gel-resolved T-ARMS-PCR assay, which is susceptible to allele-specific amplification imbalance, non-specific amplification, and genotype-calling errors [46]. Although samples with suboptimal profiles were re-amplified, the gels were independently interpreted by three investigators blinded to the participants’ clinical data, and duplicate genotyping of 10 samples, including six GA samples, showed 100% concordance, the genotype calls were not independently confirmed by Sanger sequencing or PCR-RFLP. Moreover, no-template and known-genotype positive controls were not consistently included in every experimental run.

Importantly, an AA homozygous sample previously analyzed by Chiș et al. [45] using the same rs6265 T-ARMS-PCR assay, including the same primers, master mix, and cycling protocol, showed the expected A-specific banding pattern (Figure S4), confirming that the A-specific primer was functional under these assay conditions. Nevertheless, genotype misclassification cannot be fully excluded, particularly for the rare AA genotype. Accordingly, the absence of an AA banding pattern should be regarded as an unconfirmed observation, and the genotype distribution and genotype–phenotype analyses should be interpreted as exploratory. The absence of a healthy control group precluded direct comparisons of genotype and allele frequencies between patients with AS and the general population. Consequently, the study was designed primarily to evaluate genotype–phenotype associations within the AS cohort rather than disease susceptibility.

Additionally, all 81 participants had documented positive HLA-B27 results obtained before enrolment, although HLA-B27 positivity was not an eligibility criterion. This homogeneous HLA-B27-positive cohort may limit the generalizability of the findings to the broader AS population, particularly to HLA-B27-negative patients. The specific laboratory method and testing laboratory used for the original HLA-B27 determinations could not be uniformly retrieved from the available medical records.

Given the modest sample size and limited statistical power, subtle associations between the *BDNF* Val66Met polymorphism and clinical or metabolic characteristics cannot be excluded.

The cross-sectional design does not allow causal inferences regarding the observed associations. In addition, variability in disease duration, treatment exposure, and comorbidities may have influenced several clinical and laboratory parameters. Because variables were abstracted from the latest available clinical records, measurements were not necessarily obtained at a single standardized study visit. During source-data verification, the first author identified discrepancies in waist-circumference values for six participants and corrected them according to the original medical documentation. Initial anthropometric measurements preceded BDNF genotyping; retrospective verification took place after genotype results had become available in the study database and was not blinded to genotype. The moderate BMI–waist correlation (r = 0.519) and the corresponding slope of 1.08 cm per BMI unit were weaker than generally expected in adults. Together with the source-data corrections, this pattern suggests that residual measurement error in waist circumference likely remained even after correction. Results involving waist circumference and metabolic syndrome should therefore be interpreted cautiously. Only the depression subscale (HADS-D) was available; HADS-A data were not collected. In addition, we could not document a Romanian-language validation of the HADS specifically in patients with AS. HADS-D findings should therefore be interpreted as screening results for depressive symptoms rather than psychiatric diagnoses.

The comparison between biologic and non-biologic treatment groups is subject to confounding by indication, treatment duration, previous disease severity, and patient selection and should not be interpreted as a direct estimate of treatment effect. One participant with missing biologic-therapy status was excluded from the treatment-group comparisons and adjusted regression models. Medication use for metabolic-syndrome components was not systematically incorporated into classification; therefore, the threshold-based prevalence reported here may underestimate prevalence under the full harmonized definition.

The relatively low recorded prevalence of uveitis (6/81, 7.4%) may reflect under-ascertainment inherent to retrospective medical-record review. In addition, the Benjamini–Hochberg correction was applied to the correlation analysis, whereas multiplicity was not formally controlled for the remaining secondary and exploratory analyses. Therefore, nominally significant findings from these analyses should be interpreted cautiously.

AS is a complex multifactorial disorder in which disease activity and clinical expression are shaped by a wide range of genetic, immunological, environmental, and therapeutic influences [60,61,62]. Consequently, the contribution of a single functional variant such as *BDNF* Val66Met is likely modest and may be difficult to capture in a cohort of this size.

Moreover, the biological effects of BDNF may depend on gene–gene and gene–environment interactions that were not evaluated in the present study. Disease duration, treatment exposure, physical activity, depressive symptom burden, and metabolic comorbidities may further modulate BDNF-related pathways and mask potential genotype–phenotype relationships. Finally, the impact of the Val66Met polymorphism may be more readily detectable at the level of circulating BDNF concentrations, gene expression, or within specific clinical subphenotypes, rather than across broad clinical indices such as disease activity scores or metabolic measures [63,64].

Taken together, the absence of significant associations in this cohort should not be interpreted as evidence of a lack of biological relevance but rather suggests that any effect of this polymorphism is likely modest and may require larger, well-characterized populations for detection.

Finally, this study did not include imaging findings or radiographic damage scores, which could have provided additional information regarding disease activity and structural disease burden [9,10].

Despite these limitations, the study provides novel data regarding the distribution of the *BDNF* Val66Met polymorphism and its clinical correlates in a Romanian cohort of patients with AS. Strengths of the present study include comprehensive metabolic characterization and the evaluation of clinical, inflammatory, depressive-symptom, and metabolic parameters within the same cohort.

### 4.7. Future Directions

Future studies should include a healthy control group matched for age, sex, and ethnicity to enable direct comparison of genotype and allele frequencies between patients with AS and the general population [21]. Such studies would help clarify whether the BDNF Val66Met polymorphism contributes to disease susceptibility in addition to its potential influence on disease phenotype.

Larger multicenter cohorts with adequate representation of all three genotypes, including AA homozygotes, are needed to improve statistical power and allow more robust genotype–phenotype analyses [65]. Replication of these findings in independent populations would further strengthen the evidence regarding the role of *BDNF* Val66Met in AS.

Given the cardiometabolic abnormalities observed in the present cohort, future research should explore whether *BDNF* genetic variation contributes to metabolic abnormalities and disease activity in AS, ideally using longitudinal study designs, given prior evidence linking *BDNF* variants and signaling pathways to cardiometabolic risk and metabolic dysfunction [27,64]. Such approaches may help determine whether BDNF-related pathways contribute to cardiometabolic risk in AS and whether they have potential value as biomarkers of disease outcomes.

## 5. Conclusions

In this Romanian cohort of patients with ankylosing spondylitis, the BDNF Val66Met (rs6265) polymorphism was not significantly associated with disease activity, inflammatory markers, depressive symptoms, or metabolic parameters. Given the available genotype-group sizes (n = 57 GG vs. n = 24 GA), the study had 80% power to detect standardized between-group effects of approximately Cohen’s d ≥ 0.69; therefore, smaller genotype-related associations may have remained undetected. The study provides descriptive data on rs6265 genotype distribution and the cardiometabolic profile of patients with AS, but the findings should be interpreted in the context of the modest sample size, the absence of an observed AA banding pattern without independent confirmation by sequencing or PCR-RFLP, the lack of a healthy control group, and the cross-sectional design. Larger, independent and preferably multicenter studies are needed to further clarify the potential clinical relevance of BDNF Val66Met in AS.

## Figures and Tables

**Table 1:** Demographic, therapeutic, and lifestyle characteristics of the study cohort stratified by BDNF Val66Met genotype; no AA (Met/Met) banding pattern was observed in this cohort.

Variable	Category	Total (*n* = 81)	GG Val/Val (*n* = 57)	GA Val/Met (*n* = 24)	*p*-Value
Sex, n (%)	Female	20 (24.7%)	13 (22.8%)	7 (29.2%)	0.746
	Male	61 (75.3%)	44 (77.2%)	17 (70.8%)	
Age (years), mean ± SD		53.2 ± 12.5	51.6 ± 13.0	56.8 ± 10.8	0.075
Age at diagnosis, n (%)	<30 years	46 (56.8%)	33 (57.9%)	13 (54.2%)	0.949
	≥30 years	35 (43.2%)	24 (42.1%)	11 (45.8%)	
Disease duration (years), median (IQR)		23.0 (14.0–29.0)	22.0 (14.0–28.0)	26.0 (16.8–30.8)	0.220
Current treatment, n (%)	Biologic	27 (33.3%)	18 (31.6%)	9 (37.5%)	0.797
	Non-biologic	53 (65.4%)	38 (66.7%)	15 (62.5%)	
	Missing	1(1.2%)	1(1.8%)	0(0.%)	
Smoking, n (%)	Never	40 (49.4%)	26 (45.6%)	14 (58.3%)	0.025
	Former	28 (34.6%)	18 (31.6%)	10 (41.7%)	
	Current	13 (16.0%)	13 (22.8%)	0 (0.0%)	
Alcohol consumption, n (%)	None	36 (44.4%)	23 (40.4%)	13 (54.2%)	0.741
	Occasional	10 (12.3%)	7 (12.3%)	3 (12.5%)	
	Moderate	9 (11.1%)	7 (12.3%)	2 (8.3%)	
	Regular	26 (32.1%)	20 (35.1%)	6 (25.0%)	
Physical activity, n (%)	Sedentary	56 (69.1%)	41 (71.9%)	15 (62.5%)	0.683
	Light	17 (21.0%)	11 (19.3%)	6 (25.0%)	
	Moderate-to-vigorous	8 (9.9%)	5 (8.8%)	3 (12.5%)	

Note: Percentages in the treatment rows were calculated using the full cohort denominators. The comparison of current biologic versus non-biologic treatment excluded the participant with missing treatment information (analyzed n = 80). The smoking comparison should be interpreted cautiously because no current smokers were observed in the GA group. SD, standard deviation; IQR, interquartile range.

**Table 2:** Clinical and metabolic characteristics of the cohort, stratified by *BDNF* Val66Met genotype.

Variable	Total (n = 81)	GG Val/Val (n = 57)	GA Val/Met (n = 24)	Difference (95% CI)	*p*-Value	Effect Size
CRP (mg/L) (median, IQR)	8.1 (5.6–13.3)	8.3 (6.1–13.3)	7.1 (5.1–12.7)	−0.4 (−2.8 to 1.9)	0.721	0.04
BASDAI score (mean ± SD)	3.6 ± 1.4	3.5 ± 1.4	3.7 ± 1.4	0.15 (−0.54 to 0.83)	0.668	0.11
ASDAS-CRP (mean ± SD)	2.5 ± 0.7	2.56 ± 0.66	2.49 ± 0.72	−0.07 (−0.42 to 0.28)	0.717	0.10
HADS-D score (median, IQR)	6 (5–7)	6 (4.5–7)	5.5 (5–7)	0 (−1 to 1)	0.829	0.02
Pain NRS (0–10) (median, IQR)	4 (3–6)	4 (3–6)	4 (3–5.2)	0 (−1 to 1)	0.579	0.06
Uveitis, n (%)	6 (7.4%)	4 (7.0%)	2 (8.3%)	-	1	-
Psoriasis, n (%)	5 (6.2%)	4 (7.0%)	1 (4.2%)	-	1	-
Inflammatory bowel disease, n (%)	3 (3.7%)	1 (1.8%)	2 (8.3%)	-	0.208	-
Triglycerides (mg/dL) (median, IQR)	145 (137–153)	145 (135–150)	148 (134–154)	3 (−5 to 9)	0.396	0.09
HDL-C (mg/dL) (median, IQR)	44 (38–48)	44 (38–50)	44 (40.5–47)	0 (−4 to 4)	0.967	0.01
LDL-C (mg/dL) (median, IQR)	123 (97–142)	110 (97–142)	132 (103–146)	9 (−8 to 26)	0.236	0.13
Glycemia (mg/dL)	90 (86–98)	89 (86–100)	91.5 (87.8–95.2)	1 (−5 to 6)	0.768	0.03
BMI (kg/m^2^)	27.0 ± 3.6	27.1 ± 3.4	27.0 ± 4.0	−0.11 (−2.01 to 1.79)	0.910	−0.03
Waist circumference (cm), mean ± SD	87.1 ± 7.5	86.5 ± 6.3	88.7 ± 9.7	2.23 (−2.15 to 6.62)	0.307	0.30
Systolic BP (mmHg)	134 (124–145)	134 (124–145)	134 (131–142)	0 (−7 to 8)	0.860	0.02
Diastolic BP (mmHg)	79 ± 6.3	78.5 ± 6.3	80.6 ± 6	2.22 (−0.78 to 5.23)	0.143	0.36

BP = blood pressure; CRP = C-reactive protein; ASDAS-CRP = Ankylosing Spondylitis Disease Activity Score based on C-reactive protein; BASDAI = Bath Ankylosing Spondylitis Disease Activity Index; NRS = Numerical Rating Scale; HADS-D = depression subscale of the Hospital Anxiety and Depression Scale. HADS-D data available for 79 patients (GG n = 55, GA n = 24). BMI and waist circumference showed a moderate positive Pearson correlation (r = 0.519, *p* < 0.001). In a simple linear regression model, waist circumference increased by 1.08 cm for each 1 kg/m^2^ increase in BMI.

**Table 3:** Prevalence of metabolic syndrome components in the study cohort, stratified by BDNF Val66Met genotype.

Metabolic Criterion	Total (n = 81), n (%)	GG (n = 57), n (%)	GA (n = 24), n (%)	OR for GA vs. GG (95% CI)	*p*-Value
Triglycerides ≥ 150 mg/dL	24 (29.6%)	15 (26.3%)	9 (37.5%)	1.67 (0.53–5.15)	0.424
Low HDL-C (men < 40; women < 50 mg/dL)	32 (39.5%)	20 (35.1%)	12 (50.0%)	1.84 (0.63–5.43)	0.225
Elevated systolic BP (≥130 mmHg)	57 (70.4%)	39 (68.4%)	18 (75.0%)	1.38 (0.43–4.98)	0.606
Elevated diastolic BP (≥85 mmHg)	20 (24.7%)	13 (22.8%)	7 (29.2%)	1.39 (0.40–4.56)	0.580
Elevated BP (systolic ≥ 130 and/or diastolic ≥ 85 mmHg)	61 (75.3%)	43 (75.4%)	18 (75.0%)	0.98 (0.29–3.61)	1.000
Fasting glycemia ≥ 100 mg/dL	18 (22.2%)	15 (26.3%)	3 (12.5%)	0.40 (0.07–1.66)	0.245
Elevated waist circumference (men ≥ 94; women ≥ 80 cm)	27 (33.3%)	15 (26.3%)	12 (50.0%)	2.76 (0.92–8.47)	0.069
Metabolic syndrome (≥3 of 5 criteria)	24 (29.6%)	14 (24.6%)	10 (41.7%)	2.17 (0.70–6.73)	0.182

Note: Metabolic syndrome was defined as the presence of at least three of five measured components using the harmonized Joint Interim Statement criteria. Medication use for individual metabolic components was not incorporated because these data were not systematically available. Odds ratios and 95% confidence intervals are exact conditional estimates; *p*-values were calculated using Fisher’s exact test. OR, odds ratio; CI, confidence interval; HDL-C, high-density lipoprotein cholesterol; BP, blood pressure. Waist-circumference and metabolic-syndrome results are based on the source-verified dataset. A sensitivity analysis excluding the six participants whose waist-circumference values were corrected is reported in Section 3.4.

**Table 4:** Clinical and metabolic characteristics of the analyzed cohort, stratified by current biologic-therapy status.

Variable	Analyzed Sample (n = 80)	Biologic (n = 27)	Non-Biologic (n = 53)	Difference (95% CI)	*p*-Value	Effect Size
CRP (mg/L), median (IQR)	8.1 (5.6–13.2)	5.9 (4.4–7.4)	12.0 (6.7–15.0)	−5.10 (−7.60 to −3.00)	<0.0001	0.61
ASDAS-CRP, mean ± SD	2.5 ± 0.7	1.9 ± 0.4	2.8 ± 0.6	−0.91 (−1.14 to −0.69)	<0.0001	1.74
BASDAI score, mean ± SD	3.6 ± 1.4	2.6 ± 1.1	4.1 ± 1.2	−1.52 (−2.06 to −0.98)	<0.0001	1.29
HADS-D score, median (IQR)	6.0 (5.0–7.0)	5.0 (4.2–6.0)	6.0 (5.0–7.0)	−1.00 (−2.00 to 0.00)	0.031	0.30
Pain NRS (0–10), median (IQR)	4.0 (3.0–6.0)	3.0 (1.5–3.5)	5.0 (4.0–6.0)	−2.00 (−3.00 to −2.00)	<0.0001	0.69
Age (years), median (IQR)	54.0 (47.0–61.2)	51.0 (41.0–56.0)	58.0 (48.0–66.0)	−9.00 (−14.00 to −3.00)	0.005	0.39
Disease duration (years), median (IQR)	23.0 (13.8–29.0)	22.0 (17.0–27.5)	23.0 (11.0–29.0)	1.00 (−4.00 to 6.00)	0.815	0.03
Triglycerides (mg/dL), median (IQR)	145.0 (137.8–153.2)	145.0 (138.5–149.0)	145.0 (138.0–154.0)	0.00 (−7.00 to 5.00)	0.807	0.03
HDL-C (mg/dL), median (IQR)	44.0 (38.8–48.5)	43.0 (36.5–49.5)	45.0 (40.0–47.0)	−1.00 (−6.00 to 3.00)	0.564	0.08
LDL-C (mg/dL), median (IQR)	123.0 (97.0–142.2)	123.0 (96.5–148.5)	123.0 (98.0–135.0)	3.00 (−10.00 to 20.00)	0.680	0.06
Glycemia (mg/dL), median (IQR)	90.0 (86.0–98.2)	90.0 (87.0–98.0)	89.0 (86.0–98.0)	1.00 (−4.00 to 6.00)	0.791	0.04
BMI (kg/m^2^), mean ± SD	27.0 ± 3.6	27.0 ± 3.4	27.0 ± 3.7	0.00 (−1.66 to 1.67)	0.996	0.00
Waist circumference (cm), mean ± SD	87.1 ± 7.5	86.7 ± 6.7	87.3 ± 7.9	−0.60 (−3.97 to 2.77)	0.724	0.08

Note: Treatment-status analyses included 80 participants. One participant with missing treatment information was excluded. Biologic therapy refers to current treatment status and does not necessarily indicate biologic monotherapy. HADS-D data were available for 78 of the 80 participants included in this analysis (26 receiving biologic therapy and 52 receiving non-biologic treatment). For normally distributed variables, the difference represents the mean difference, and the effect size represents the absolute value of Cohen’s d. For non-normally distributed variables, the difference represents the Hodges–Lehmann location difference and the effect size represents the absolute rank-biserial correlation. CI, confidence interval; IQR, interquartile range; SD, standard deviation; CRP, C-reactive protein; ASDAS-CRP, Ankylosing Spondylitis Disease Activity Score using CRP; BASDAI, Bath Ankylosing Spondylitis Disease Activity Index; HADS-D, depression subscale of the Hospital Anxiety and Depression Scale; NRS, Numerical Rating Scale; HDL-C, high-density lipoprotein cholesterol; LDL-C, low-density lipoprotein cholesterol; BMI, body mass index.

**Table 5:** Selected nominally significant Spearman rank correlations in the study cohort.

Variable 1	Variable 2	n	Spearman r	*p*-Value	BH q-Value
Pain NRS	BASDAI score	81	0.754	<0.0001	<0.0001
Pain NRS	HADS-D score	79	0.378	0.0006	0.0030
Pain NRS	CRP	81	0.372	0.0006	0.0030
BASDAI score	HADS-D score	79	0.536	<0.0001	<0.0001
Age	Age at diagnosis	81	0.467	<0.0001	<0.0001
Systolic BP	BMI	81	0.588	<0.0001	<0.0001

Note: Benjamini–Hochberg correction was applied across all 28 pairwise correlation tests. Only associations with q < 0.05 are shown. The correlation between age and age at diagnosis is structurally expected and should not be interpreted as an independent clinical finding. Complete results are provided in Supplementary Table S2. NRS, Numerical Rating Scale; BASDAI, Bath Ankylosing Spondylitis Disease Activity Index; HADS-D, depression subscale of the Hospital Anxiety and Depression Scale; CRP, C-reactive protein; BP, blood pressure; BMI, body mass index.

## Data Availability

The data supporting the findings of this study are available from the corresponding author upon reasonable request, subject to applicable ethical and privacy restrictions. A data-free analysis script is provided as Supplementary File S1 for methodological transparency. The script is syntactically valid but has not been validated against the final individual-level dataset; therefore, complete reproduction of all numerical results reported in the manuscript from this script has not been established.

## Supplementary Materials

The following supporting information can be downloaded at https://www.mdpi.com/article/10.3390/cimb48101018/s1.

## Abbreviations

The following abbreviations are used in this manuscript:

AS	Ankylosing spondylitis
ASAS	Assessment of SpondyloArthritis International Society
ASDAS	Ankylosing Spondylitis Disease Activity Score
BASDAI	Bath Ankylosing Spondylitis Disease Activity Index
BDNF	Brain-derived neurotrophic factor
BMI	Body mass index
BP	Blood pressure
CI	Confidence interval
CRP	C-reactive protein
ESR	Erythrocyte sedimentation rate
EULAR	European Alliance of Associations for Rheumatology
HADS-D	Depression subscale of the Hospital Anxiety and Depression Scale
HDL-cholesterol	High-Density Lipoprotein Cholesterol
HLA	Human Leukocyte Antigen
HWE	Hardy–Weinberg equilibrium
IL	Interleukin
LDL-cholesterol	Low-Density Lipoprotein Cholesterol
IQR	Interquartile range
mRNA	Messenger RNA
MHC	Major Histocompatibility Complex
NRS	Numerical Rating Scale
NSAIDs	Nonsteroidal anti-inflammatory drugs
PGA	Patient Global Assessment
SD	Standard deviation
SpA	Spondyloarthritis
T-ARMS-PCR	Tetra-primer Amplification Refractory Mutation System PCR
TNF-α	Tumor Necrosis Factor-α
Val66Met	Valine-to-methionine substitution at codon 66
