# Supplementary material for: BDNF Val66Met (rs6265) in Ankylosing Spondylitis: A Descriptive Genotype–Phenotype Study in a Romanian Cohort
Source: Curr Issues Mol Biol. 2026 Oct 2;48(10):1018. doi: 10.3390/cimb48101018 (PMC13703505; doi:10.3390/cimb48101018)
Supplement: Supplementary file 1 [file cimb-48-01018-s001.zip › cimb-4524689-supplementary.pdf]

Supplementary Table S3. STREGA Checklist

BDNF Val66Met (rs6265) in Ankylosing Spondylitis: A Descriptive Genotype–Phenotype Study in a Romanian Cohort

Completed per Little J, Higgins JP, Ioannidis JP, et al. Strengthening the reporting of genetic association studies (STREGA): an extension of the STROBE statement. PLoS Med. 2009;6(2):e1000022.

Section/Item	STREGA #	Recommendation	Where addressed in the manuscript
Title and abstract	1	State the study design, exposure (BDNF Val66Met genotype) and outcome (disease activity, cardiometabolic parameters) in the title/abstract.	Title revised to reflect a descriptive genotype-distribution and cardiometabolic-characterization study; Abstract states design (single-center, cross-sectional), cohort, exposure, and outcomes.
Introduction — background/rationale	2	Explain scientific background and rationale.	Introduction (Section 1) provides background on AS, BDNF biology, Val66Met function, and rationale for this study.
Introduction — objectives	3	State specific objectives, including any prespecified hypotheses.	Study reframed as descriptive/exploratory; ASDAS-CRP designated as the primary outcome (Introduction and Section 2.4); other comparisons labeled secondary/exploratory.
Methods — study design	4	Present key elements of study design early.	Section 2.1 states the cross-sectional design and recruitment period.
Methods — setting	5	Describe setting, locations, and relevant dates.	Section 2.1: Rheumatology Department, Bihor County Emergency Clinical Hospital, Oradea, Romania, 1 March–30 June 2026.
Methods — participants	6	Eligibility criteria, sources, methods of selection; for genetic association studies, describe how population stratification was assessed and controlled.	Section 2.1: modified New York criteria for AS. Population stratification was not directly assessed (single-center, ethnically homogeneous Romanian cohort); this is noted as a limitation (Section 4.6).

Section/Item	STREGA #	Recommendation	Where addressed in the manuscript
Methods — variables	7	Define all outcomes, exposures, predictors, confounders, effect modifiers; give diagnostic criteria if applicable.	Section 2.2 defines all clinical, laboratory, and anthropometric variables; Section 2.3 defines the genetic exposure (BDNF Val66Met/rs6265).
Methods — data sources/measurement — genotyping (M1)	8	Genotyping method, source and storage of DNA, genotyping platform, call rate, error rates, and discordance checks.	Section 2.3 describes T-ARMS-PCR genotyping. Section 2.3: primer sequences, PCR conditions, and Mg2+ concentration (Supplementary Table S1); Section 3.2: call rate (95.1% first-pass, 100% final after re-amplification of four samples) and blinded duplicate genotyping of 10/81 samples (12.3%; six GA and four GG), with 100% concordance. Genotype calls were not independently confirmed by Sanger sequencing or PCR-RFLP; this is stated explicitly as a limitation (Section 4.6).
Methods — data sources/measurement — HWE (M2)	9	State whether HWE was assessed, and in which population.	Section 3.2: HWE assessed by exact test in the full case cohort (no control group); explicitly noted that departure from HWE in a case-only design cannot distinguish genuine association from genotyping error, so HWE compliance is not presented as evidence of genotyping quality.
Methods — data sources/measurement — QC (M3)	10	Describe quality control methods.	Section 2.3/3.2: genotype calls were made independently by three investigators blinded to clinical data; samples with ambiguous profiles were re-amplified before a final call; 10 samples (six GA and four GG) were re-genotyped in a blinded manner, with 100% concordance. No-template and known-genotype positive controls were not consistently included in every run; this is stated as a limitation in Section 4.6.
Methods — bias	11	Describe efforts to address potential sources of bias.	Section 4.6 (Limitations) discusses retrospective chart abstraction, absence of a control group, and potential measurement variability (e.g., waist circumference) as sources of bias.

Section/Item	STREGA #	Recommendation	Where addressed in the manuscript
Methods — study size	12	Explain how study size was arrived at.	Section 4.6 and the post-hoc power calculation (80% power to detect Cohen's d ≥ 0.69 given n=57 vs. n=24) describe the achieved sample size and its limitations; the study size reflects all eligible patients recruited during the study period rather than a prespecified target.
Methods — quantitative variables	13	Explain how quantitative variables were handled; describe grouping.	Section 2.4: continuous variables reported as mean±SD or median (IQR) depending on distribution; genotype compared as GG vs. G/A (no A/A carriers observed).
Methods — statistical methods (m8)	14	Describe all statistical methods, including those for confounding, subgroups, and interactions; state software used.	Section 2.4 describes the assessment of data distribution, between-group comparisons, effect-size estimates with 95% confidence intervals, Spearman rank correlations, Hardy–Weinberg equilibrium testing, and exploratory multivariable linear and logistic regression. The Benjamini–Hochberg false discovery rate procedure was applied across the 28 pairwise correlation tests. Statistical software and version information are also reported.
Methods — statistical methods — subgroup/sensitivity	15	Describe any sensitivity analyses.	Sex-stratified comparisons and adjusted multivariable regression models were performed as secondary exploratory analyses and are reported in Sections 3.1 and 3.5. No additional formal sensitivity analyses were performed.
Results — participants	16	Report numbers of individuals at each stage of the study.	Section 3.1/2.1: Section 2.1: 81 patients recruited consecutively during the study period, all fulfilling the modified New York criteria; DNA from all 81 was genotyped and all 81 yielded a valid genotype (four samples re-amplified after a suboptimal first attempt), with no attrition between recruitment and final analysis.
Results — descriptive data by genotype	17	Give characteristics of study participants and information on	Tables 1–3 report demographic, clinical, and metabolic characteristics stratified by BDNF genotype (GG vs. G/A).

Section/Item	STREGA #	Recommendation	Where addressed in the manuscript
		exposures, stratified by genotype/allele.	
Results — outcome data	18	Report numbers of outcome events or summary measures.	Tables 2–4 and Section 3.3–3.5 report all outcome comparisons with effect sizes and 95% CIs.
Results — main results	19	Give unadjusted and adjusted estimates with precision (e.g., 95% CI); specify confounders adjusted for.	Multivariable linear regression models for the primary outcome, ASDAS-CRP, and the secondary outcome, BASDAI, are reported with regression coefficients and 95% confidence intervals. The models were adjusted for genotype, age, sex, disease duration, current biologic therapy, and smoking status. The exploratory logistic regression model for metabolic syndrome is also reported, together with its limited events-per-variable ratio.
Results — HWE	20	Report distribution of genotypes and results of HWE testing.	Section 3.2 and Figure 1: observed vs. HWE-expected genotype frequencies (57 GG, 24 G/A, 0 A/A vs. 58.8/20.4/1.8 expected), exact test p=0.198.
Results — other analyses	21	Report other analyses done (subgroups, interactions, sensitivity analyses).	Sex-stratified analyses (Section 3.1), exploratory analyses by biologic-therapy status and adjusted regression models (Section 3.5), and correlation analyses (Section 3.6) are reported. Complete results for all 28 pairwise correlations, including Benjamini–Hochberg q-values, are provided in Supplementary Table S2.
Discussion — key results	22	Summarize key results with reference to study objectives.	Section 4 opens each subsection with a summary of the relevant findings relative to the study's descriptive/exploratory aim.
Discussion — limitations	23	Discuss limitations, sources of bias/imprecision, and multiplicity of analyses.	Section 4.6 discusses sample size, absence of AA homozygotes, lack of independent sequencing or PCR-RFLP confirmation, absence of a healthy control group, the exclusively HLA-B27-positive cohort, cross-sectional design, retrospective data abstraction, potential under-

Section/Item	STREGA #	Recommendation	Where addressed in the manuscript
			ascertainment, and unavailable medication data. Multiplicity in the correlation analysis was addressed using the Benjamini–Hochberg procedure; the remaining analyses are explicitly described as exploratory.
Discussion — interpretation	24	Give cautious overall interpretation considering objectives, limitations, and multiplicity of analyses.	Section 4.2, 4.5, 4.6: findings interpreted as descriptive/exploratory; power limitations and EPV caveats stated explicitly alongside each null result.
Discussion — generalizability	25	Discuss generalizability of the results.	Section 4.6/4.7 note the single-center, Romanian, HLA-B27-positive cohort and call for replication in larger, multicenter, multi-ethnic populations.
Other information — funding	26	Give source of funding and role of funders.	Funding statement: APC funded by University of Oradea; no role in study design, analysis, or interpretation stated.
Other information — replication	27	State whether replication of the findings was attempted, and discuss.	No independent replication cohort was available; Section 4.7 (Future Directions) calls for replication in larger, multicenter cohorts.

Supplementary material

regarding the genotyping for the manuscript "BDNF Val66Met (rs6265) in Ankylosing Spondylitis: A Descriptive Genotype-Phenotype Study in a Romanian Cohort"

The BDNF rs6265 (Val66Met) polymorphism was genotyped by tetra-primer amplification-refractory mutation system PCR (T-ARMS-PCR), using the four primers reported by Wang et al. (2015), with the outer primers amplifying a common fragment and the allele-specific inner primers carrying a deliberate mismatch at the third position from the 3′ terminus to enhance allelic discrimination.

Amplification was performed with the 5× FIREPol Ready to load Master Mix with 7.5 mM $MgCl_2$ (Solis BioDyne, Tartu, Estonia), a *ready-to-use* mixture containing the DNA polymerase, reaction buffer, dNTPs and $MgCl_2$.

A touchdown PCR protocol was used for rs6265 genotyping. Initial denaturation was performed at 94°C for 3 min, followed by 30 cycles consisting of denaturation at 94°C for 30 s, annealing for 30 s, and extension at 72°C for 30 s. The annealing temperature decreased from 63°C to 57°C by 0.3°C per cycle during cycles 1–20 and was maintained at 57°C during cycles 21–30. A final extension was performed at 72°C for 5 min, followed by a hold at 4°C.

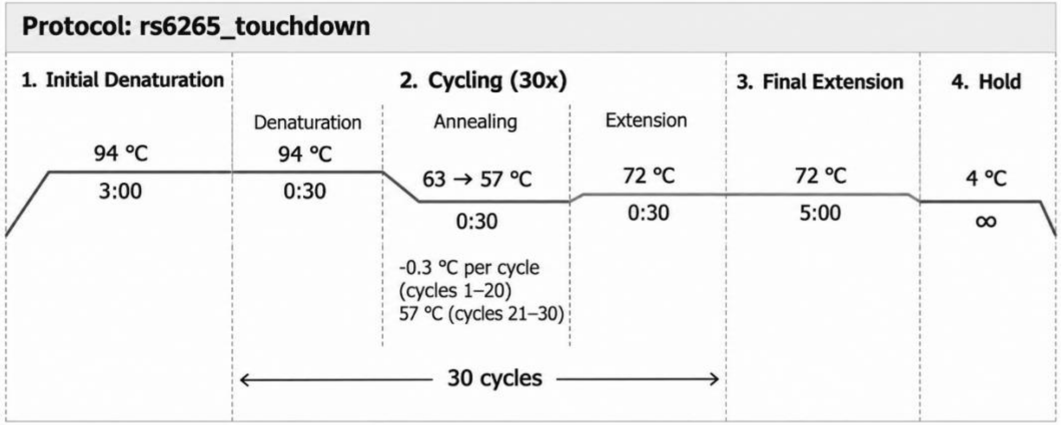

Supplementary Figure S1. Schematic representation of the touchdown PCR cycling conditions, including annealing temperatures, used for genotyping the BDNF rs6265 SNP, based on the protocol described by Wang et al. (2015) and performed using a Bio-Rad CFX96 thermocycler.

Each 20 μL reaction contained 2 μL of genomic DNA, 4 μL of 5× FIREPol Ready to Load Master Mix (Solis BioDyne, Tartu, Estonia), 12 μL of nuclease-free water, 0.25 μL of each outer primer (OF and OR; 10 μM stock concentration), and 0.75 μL of each inner allele-specific primer (IF and IR; 10 μM stock concentration). This corresponded to final primer concentrations of 0.125 μM for each outer primer and 0.375 μM for each inner primer, with a 1:3 outer-to-inner primer ratio, as recommended for tetra-primer ARMS-PCR (Ye et al., 2001). The final $MgCl_2$ concentration in the reaction was 1.5 mM.

PCR amplification was performed using a Bio-Rad CFX96 thermal cycler (Bio-Rad Laboratories). The outer primers generated the 406-bp common amplification-control product, whereas the inner primers generated the allele-specific products. PCR products were resolved on a 3% agarose gel, stained with Midori Green (NIPPON Genetics Europe GmbH, Düren, Germany), and visualized under UV light. The expected Val- and Met-specific products were 253 bp and 203 bp, respectively.

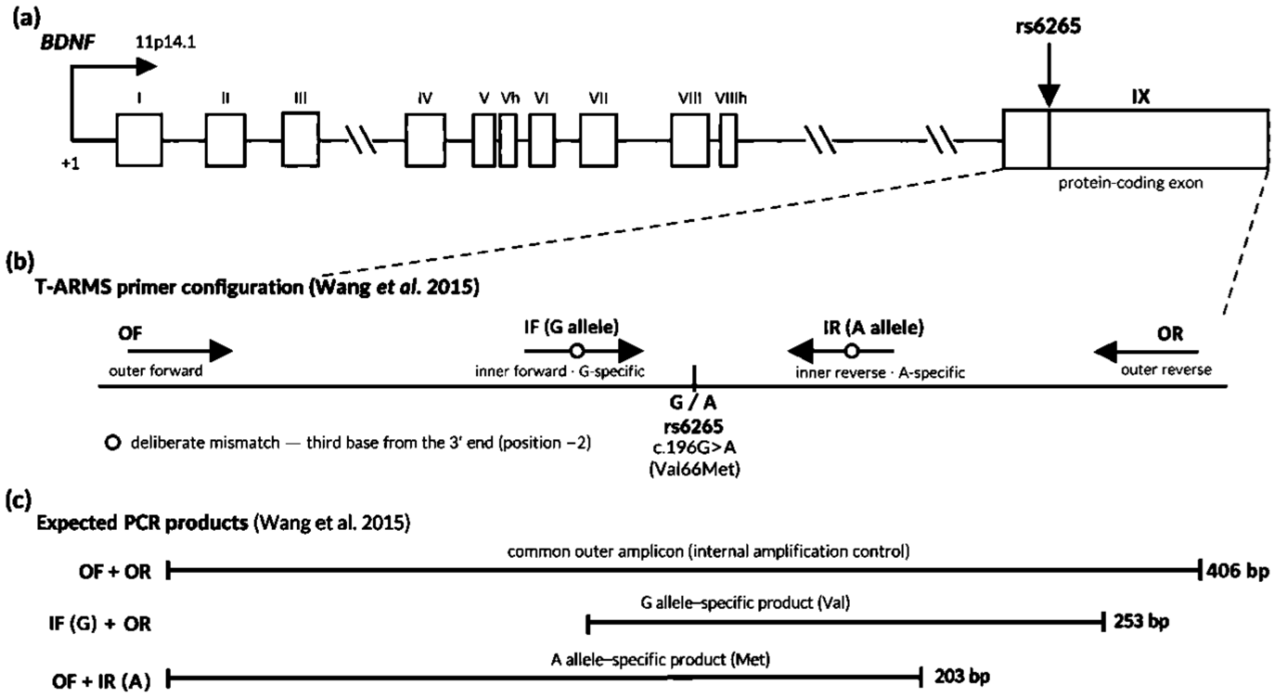

Supplementary Figure S2. Schematic representation of the BDNF gene, rs6265 location, T-ARMS-PCR primer configuration, and expected amplification products. (a) Position of rs6265 (c.196G>A; p.Val66Met) within the protein-coding exon of BDNF; the gene structure is not drawn to scale. (b) Four-primer configuration: outer forward (OF), G-specific inner forward [IF(G)], A-specific inner reverse [IR(A)], and outer reverse (OR). The inner primers contain a deliberate mismatch near the 3′ end to enhance allele discrimination (Ye et al., 2001; Wang et al., 2015). (c) Expected amplification products: a 406-bp common outer-primer product generated by OF and OR, a 253-bp G/Val-specific product generated by IF(G) and OR, and a 203-bp A/Met-specific product generated by OF and IR(A). The schematic was created based on the protocol described by Wang et al. (2015). **Common outer control amplicon = 406 bp = OF + OR.**

In the present analysis, the 406-bp common outer-primer product served as an internal amplification control. The G/Val- and A/Met-specific products were 253 bp and 203 bp, respectively. Accordingly, GG samples showed bands at 406 and 253 bp, GA samples showed bands at 406, 253, and 203 bp, and AA samples would be identified by bands at 406 and 203 bp. No AA pattern was observed in the present cohort.

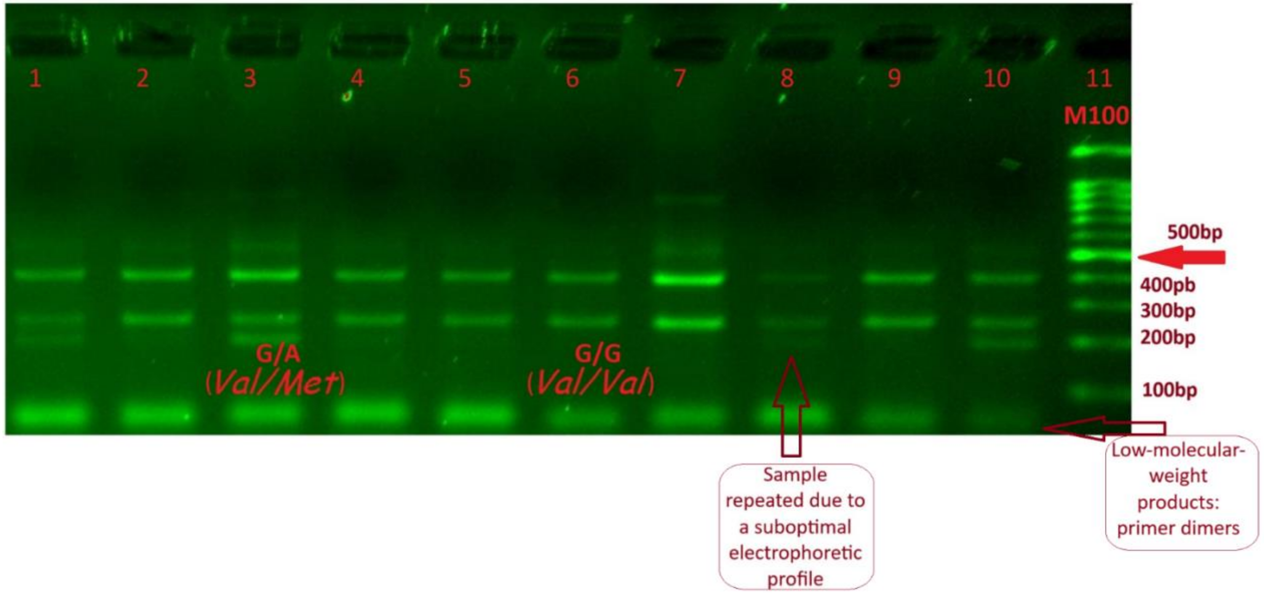

Supplementary Figure S3. Illustration of *BDNF* Val66Met PCR products separated on a 3% agarose gel, stained with Midori Green, and visualized under UV light. M100: 100-bp DNA molecular weight marker.

Lane 11 contains a 100-bp DNA molecular weight marker. Lane 8 shows a suboptimal electrophoretic profile; this sample was subsequently re-amplified before a final genotype was assigned. Lanes 1, 3, and 10 show the GA (Val/Met) genotype, whereas lanes 2, 4–7, and 9 show the GG (Val/Val) genotype. Genotypes were assigned according to the T-ARMS-PCR banding pattern: the 406-bp outer-primer product served as an internal amplification control, homozygous samples showed one allele-specific product, and heterozygous samples showed both allele-specific products. Only GG and GA patterns were observed in the present cohort; no AA pattern was identified. Bands below 100 bp represent low-molecular-weight products consistent with primer dimers.

Of the 81 samples analyzed, 77 yielded clear genotyping patterns on the first analysis, while four showed suboptimal agarose gel electrophoretic profiles (an example is shown in lane 8 of Supplementary Figure S3) and were therefore repeated; after repeat analysis, all 81 samples were successfully genotyped with clear electrophoretic patterns. To minimize genotyping error, all agarose gels were read blind by three independent investigators. To assess genotyping reproducibility, ten randomly selected samples (12.3% of the cohort) were re-genotyped in a blinded manner; six were heterozygous (GA) and four were homozygous (GG). All repeated genotype calls were concordant with the initial results, corresponding to a concordance rate of 100%. Genotypes were assigned without knowledge of the participants' clinical status.

Limitations: No-template controls and positive controls with known genotypes were not consistently included in every experimental run, and the genotype calls were not independently confirmed by sequencing or PCR-RFLP.

In two of our previous, larger studies conducted in Romanian non-rheumatological populations (n = 1,124 and n = 266), the AA (Met/Met) genotype occurred at frequencies of 4.00% and 5.26%, respectively (Vulturar et al., 2016; Bîlc et al., 2018). Assuming these frequencies, the probabilities of observing no AA homozygotes in a cohort of 81 individuals would be approximately 3.7% and 1.3%, respectively. Thus, the absence of AA homozygotes in the present cohort represents a borderline or uncommon finding. Although it may have occurred by chance, it is also compatible with, but does not prove, undetected genotype misclassification.

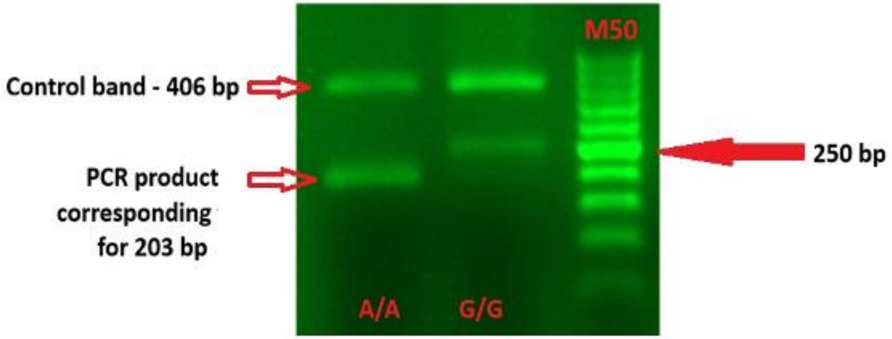

Supplementary Figure S4. Representative AA (Met/Met) T-ARMS-PCR profile obtained in a previous study. The AA sample was analyzed using the same rs6265 T-ARMS-PCR assay, including the same primers, master mix, and cycling protocol as those used for the present cohort, demonstrating that the A-specific primer was functional under these assay conditions (Chiș et al., 2024). Lane M, 50-bp DNA molecular weight marker.

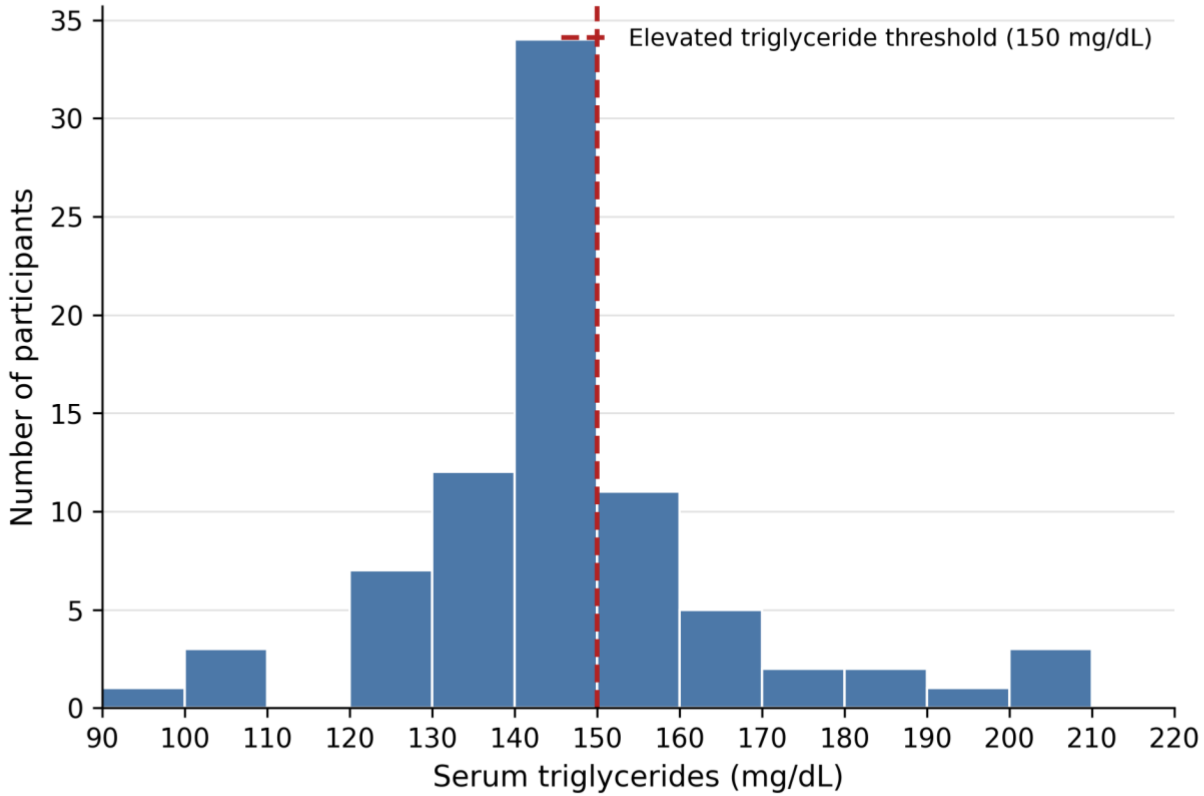

Supplementary Figure S5. Distribution of serum triglyceride concentrations in the study cohort. The histogram shows the distribution of serum triglyceride concentrations among all 81 participants (median, 145 mg/dL; IQR, 137–153 mg/dL; range, 94–208 mg/dL). The dashed vertical line indicates the threshold for elevated triglycerides (≥150 mg/dL). Individual values were re-verified against the study database.

Supplementary Table S1. Primer sequences and final concentrations used for T-ARMS-PCR genotyping of BDNF rs6265.

Primer	Sequence (5′→3′)	Final concentration
rs6265_TARMS_OF (outer forward)	CCTACAGTTCCACCAGGTGAGAAG	0.125 μM
rs6265_TARMS_OR (outer reverse)	GACCCTCATGGACATGTTTGC	0.125 μM
rs6265_TARMS_IF (inner forward)	GGCTGACACTTTCGAAC**c**CA	0.375 μM
rs6265_TARMS_IR (inner reverse)	GGTCCTCATCCAACAGCTCTTCTAT**a**AC	0.375 μM

Note. Lowercase bold nucleotides in the inner primers indicate deliberate mismatches introduced near the 3′ end to enhance allele specificity (Ye et al., 2001; Wang et al., 2015).

Supplementary Table S2. Pairwise Spearman rank correlations among clinical, inflammatory, and metabolic variables in the study cohort

Variable 1	Variable 2	n	Spearman r	p-value	BH q-value
**Pain NRS**	**BASDAI score**	**81**	**0.754**	**<0.0001**	**<0.0001**
**Pain NRS**	**HADS-D score**	**79**	**0.378**	**0.0006**	**0.0030**
**Pain NRS**	**CRP**	**81**	**0.372**	**0.0006**	**0.0030**
Pain NRS	Age	81	0.140	0.2116	0.3292
Pain NRS	Age at diagnosis	81	0.127	0.2597	0.3827
Pain NRS	Systolic BP	81	0.102	0.3650	0.4307
Pain NRS	BMI	81	−0.079	0.4841	0.5370
**BASDAI score**	**HADS-D score**	**79**	**0.536**	**<0.0001**	**<0.0001**
BASDAI score	CRP	81	0.233	0.0367	0.1058
BASDAI score	Age	81	0.231	0.0380	0.1058
BASDAI score	Age at diagnosis	81	0.227	0.0416	0.1058
BASDAI score	Systolic BP	81	0.167	0.1358	0.2717
BASDAI score	BMI	81	0.006	0.9575	0.9575
HADS-D score	CRP	79	0.121	0.2872	0.3931
HADS-D score	Age	79	0.007	0.9497	0.9575
HADS-D score	Age at diagnosis	79	0.163	0.1524	0.2765
HADS-D score	Systolic BP	79	−0.102	0.3692	0.4307
HADS-D score	BMI	79	−0.180	0.1133	0.2441
CRP	Age	81	0.156	0.1642	0.2765
CRP	Age at diagnosis	81	0.230	0.0386	0.1058
CRP	Systolic BP	81	0.114	0.3089	0.3931
CRP	BMI	81	0.076	0.4986	0.5370
**Age**	**Age at diagnosis**	**81**	**0.467**	**<0.0001**	**<0.0001**
Age	Systolic BP	81	0.269	0.0153	0.0614
Age	BMI	81	0.205	0.0662	0.1545
Age at diagnosis	Systolic BP	81	0.155	0.1679	0.2765
Age at diagnosis	BMI	81	0.117	0.2997	0.3931

**Systolic BP**	**BMI**	**81**	**0.588**	**<0.0001**	**<0.0001**

Note: The Benjamini–Hochberg procedure was applied across all 28 pairwise tests. Associations remaining statistically significant after correction ($q < 0.05$) are shown in bold. HADS-D data were available for 79 participants; all other analyses included 81 participants. The correlation between age and age at diagnosis is structurally expected and should not be interpreted as an independent clinical association. NRS, Numerical Rating Scale; BASDAI, Bath Ankylosing Spondylitis Disease Activity Index; HADS-D, depression subscale of the Hospital Anxiety and Depression Scale; CRP, C-reactive protein; BP, blood pressure; BMI, body mass index.

References

Wang, C.K.; Xu, M.S.; Ross, C.J.; Lo, R.; Procyshyn, R.M.; Vila-Rodriguez, F.; White, R.F.; Honer, W.G.; Barr, A.M. Development of a cost-efficient novel method for rapid, concurrent genotyping of five common single nucleotide polymorphisms of the brain derived neurotrophic factor (BDNF) gene by tetra-primer amplification refractory mutation system. Int. J. Methods Psychiatr. Res. **2015**, 24(3), 235–244. https://doi.org/10.1002/mpr.1475

Ye, S.; Dhillon, S.; Ke, X.; Collins, A.R.; Day, I.N.M. An efficient procedure for genotyping single nucleotide polymorphisms. Nucleic Acids Res. **2001**, 29(17), e88. https://doi.org/10.1093/nar/29.17.e88

Chiș, A.; Oltean, L.-E.; Bîlc, M.; Vulturar, R.; Șoflău, R.; David, D.; Szentágotai-Tătar, A.; Miu, A.C. Gene–Environment Interactions in Irrational Beliefs: The Roles of Childhood Adversity and Multiple Candidate Genes. *Int. J. Mol. Sci.* **2024**, *25*, 4206. https://doi.org/10.3390/ijms25084206

Pompanon, F.; Bonin, A.; Bellemain, E.; Taberlet, P. Genotyping errors: Causes, consequences and solutions. Nat. Rev. Genet. **2005**, 6, 847–859. https://doi.org/10.1038/nrg1707

Vulturar, R.; Chiş, A.; Hambrich, M.; Kelemen, B.; Ungureanu, L.; Miu, A.C. Allelic distribution of BDNF Val66Met polymorphism in healthy Romanian volunteers. Transl. Neurosci. **2016**, 7(1), 31–34. https://doi.org/10.1515/tnsci-2016-0006

Bîlc, M.I.; Vulturar, R.; Chiș, A.; Buciuman, M.; Nuţu, D.; Bunea, I.; Szentágotai-Tătar, A.; Miu, A.C. Childhood trauma and emotion regulation: The moderator role of BDNF Val66Met. Neurosci. Lett. **2018**, 685, 7–11. https://doi.org/10.1016/j.neulet.2018.07.018

```
#!/usr/bin/env python3
"""Reproducible analysis code for the BDNF Val66Met ankylosing
spondylitis study.

The script contains no participant-level observations or
identifiers. It reads the
private study dataset supplied by the investigators and writes
aggregate results.

Expected input columns (current database labels):
Pacient, Gen, Varsta, An_diagnostic, Durata_boala_ani,
BDNF_Val66Met,
BASDAI, Scala_durere, CRP_mgL, HADS, Tratament, Tratament_actual,
Fumat,
Greutate_kg, Inaltime_cm, Circumf_abd_cm, TAS_mmHg, TAD_mmHg,
Trigliceride_mgdl, HDL_mgdl, LDL_mgdl, Glicemie_mgdl, ASDAS_CRP.

For the prespecified source-correction sensitivity analysis, the
private analysis
copy must also contain a Boolean or 0/1 column named
Waist_corrected. Exactly the
six source-verified waist records should be marked 1. This flag is
non-identifying.
"""

from __future__ import annotations

import argparse
from pathlib import Path

import numpy as np
import pandas as pd
from scipy import stats
from scipy.optimize import minimize
from scipy.special import expit
from scipy.stats.contingency import odds_ratio


REQUIRED = [
    "Gen", "Varsta", "An_diagnostic", "Durata_boala_ani",
"BDNF_Val66Met",
    "BASDAI", "Scala_durere", "CRP_mgL", "HADS", "Tratament",
    "Tratament_actual", "Fumat", "Greutate_kg", "Inaltime_cm",
    "Circumf_abd_cm", "TAS_mmHg", "TAD_mmHg", "Trigliceride_mgdl",
    "HDL_mgdl", "LDL_mgdl", "Glicemie_mgdl", "ASDAS_CRP",
]


def parse_args() -> argparse.Namespace:
    parser = argparse.ArgumentParser()
    parser.add_argument("input", type=Path, help="Private .xlsx
or .csv study dataset")
    parser.add_argument("--sheet", default="Date_81pac", help="Excel
worksheet name")
```

```
    parser.add_argument("--header-row", type=int, default=2,
                        help="One-based row containing variable
names in Excel")
    parser.add_argument("--output-dir", type=Path,
default=Path("analysis_output"))
    return parser.parse_args()


def read_dataset(path: Path, sheet: str, header_row: int) ->
pd.DataFrame:
    if path.suffix.lower() == ".csv":
        df = pd.read_csv(path)
    else:
        df = pd.read_excel(path, sheet_name=sheet, header=header_row
- 1)
    df = df.dropna(how="all")
    missing = [c for c in REQUIRED if c not in df.columns]
    if missing:
        raise ValueError(f"Missing required columns: {missing}")
    if len(df) != 81:
        raise ValueError(f"Expected 81 participants; found
{len(df)}")
    return df.copy()


def derive_variables(df: pd.DataFrame) -> pd.DataFrame:
    d = df.copy()
    d["genotype"] = d["BDNF_Val66Met"].astype(str).str.replace("/",
"", regex=False)
    if set(d["genotype"]) != {"GG", "GA"}:
        raise ValueError("Expected only GG and GA genotype calls")
    d["female"] = (d["Gen"] == "F").astype(int)
    d["BMI"] = d["Greutate_kg"] / (d["Inaltime_cm"] / 100.0) ** 2
    d["age_at_diagnosis"] = d["An_diagnostic"] - (2026 -
d["Varsta"])
    d["current_smoker"] = (d["Fumat"] == 2).astype(int)
    d["biologic"] = np.where(
        d["Tratament_actual"].astype(str).str.upper().eq("BIOL"), 1,
        np.where(d["Tratament"].notna(), 0, np.nan),
    )
    d["waist_high"] = np.where(
        d["Gen"].eq("M"), d["Circumf_abd_cm"].ge(94),
d["Circumf_abd_cm"].ge(80)
    )
    d["waist_high_ncep"] = np.where(
        d["Gen"].eq("M"), d["Circumf_abd_cm"].ge(102),
d["Circumf_abd_cm"].ge(88)
    )
    d["tg_high"] = d["Trigliceride_mgdl"].ge(150)
    d["hdl_low"] = np.where(
        d["Gen"].eq("M"), d["HDL_mgdl"].lt(40), d["HDL_mgdl"].lt(50)
    )
    d["bp_high"] = d["TAS_mmHg"].ge(130) | d["TAD_mmHg"].ge(85)
    d["glucose_high"] = d["Glicemie_mgdl"].ge(100)
```

```
    other = d[["tg_high", "hdl_low", "bp_high",
"glucose_high"]].astype(int).sum(axis=1)
    d["metabolic_syndrome"] =
other.add(d["waist_high"].astype(int)).ge(3)
    d["metabolic_syndrome_ncep"] =
other.add(d["waist_high_ncep"].astype(int)).ge(3)
    return d


def hwe_exact(obs_hets: int, obs_hom1: int, obs_hom2: int) -> float:
    """Exact Hardy-Weinberg test following Wigginton et al. 2005."""
    if obs_hom1 < 0 or obs_hom2 < 0 or obs_hets < 0:
        raise ValueError("Genotype counts must be non-negative")
    obs_homc, obs_homr = sorted((obs_hom1, obs_hom2))
    rare = 2 * obs_homr + obs_hets
    genotypes = obs_hets + obs_homc + obs_homr
    probs = np.zeros(rare + 1)
    mid = int(rare * (2 * genotypes - rare) / (2 * genotypes))
    if (rare & 1) != (mid & 1):
        mid += 1
    probs[mid] = 1.0
    total = 1.0
    curr_hets = mid
    curr_homr = (rare - mid) // 2
    curr_homc = genotypes - curr_hets - curr_homr
    while curr_hets >= 2:
        prob = probs[curr_hets] * curr_hets * (curr_hets - 1)
        prob /= 4.0 * (curr_homr + 1) * (curr_homc + 1)
        probs[curr_hets - 2] = prob
        total += prob
        curr_hets -= 2
        curr_homr += 1
        curr_homc += 1
    curr_hets = mid
    curr_homr = (rare - mid) // 2
    curr_homc = genotypes - curr_hets - curr_homr
    while curr_hets <= rare - 2:
        prob = probs[curr_hets] * 4.0 * curr_homr * curr_homc
        prob /= (curr_hets + 2) * (curr_hets + 1)
        probs[curr_hets + 2] = prob
        total += prob
        curr_hets += 2
        curr_homr -= 1
        curr_homc -= 1
    probs /= total
    observed = probs[obs_hets]
    return float(min(1.0, probs[probs <= observed + 1e-12].sum()))


def conditional_or(table: np.ndarray) -> dict[str, float]:
    result = odds_ratio(table, kind="conditional")
    ci = result.confidence_interval(confidence_level=0.95)
    p = stats.fisher_exact(table, alternative="two-sided").pvalue
    return {"or": result.statistic, "ci_low": ci.low, "ci_high":
```

```
ci.high, "p": p}


def binary_genotype_table(d: pd.DataFrame, outcome: str) ->
np.ndarray:
    # Rows are GA, GG; columns are criterion present, absent.
    out = d[outcome].astype(bool)
    ga = d["genotype"].eq("GA")
    return np.array([
        [(ga & out).sum(), (ga & ~out).sum()],
        [(~ga & out).sum(), (~ga & ~out).sum()],
    ], dtype=int)


def genotype_binary_results(d: pd.DataFrame) -> pd.DataFrame:
    records = []
    outcomes = ["tg_high", "hdl_low", "bp_high", "glucose_high",
"waist_high",
                "metabolic_syndrome"]
    for outcome in outcomes:
        table = binary_genotype_table(d, outcome)
        estimate = conditional_or(table)
        records.append({
            "outcome": outcome,
            "GA_n": int(table[0, 0]), "GA_total":
int(table[0].sum()),
            "GG_n": int(table[1, 0]), "GG_total":
int(table[1].sum()),
            **estimate,
        })
    return pd.DataFrame(records)


def correlations(d: pd.DataFrame) -> pd.DataFrame:
    variables = {
        "Pain NRS": "Scala_durere", "BASDAI": "BASDAI", "HADS-D":
"HADS",
        "CRP": "CRP_mgL", "Age": "Varsta", "Age at diagnosis":
"age_at_diagnosis",
        "Systolic BP": "TAS_mmHg", "BMI": "BMI",
    }
    records = []
    keys = list(variables)
    for i, left in enumerate(keys):
        for right in keys[i + 1:]:
            pair = d[[variables[left], variables[right]]].dropna()
            r, p = stats.spearmanr(pair.iloc[:, 0], pair.iloc[:, 1])
            records.append({"variable_1": left, "variable_2": right,
"n": len(pair),
                            "spearman_r": r, "p_value": p})
    result = pd.DataFrame(records)
    p = result["p_value"].to_numpy()
    order = np.argsort(p)
    ranked = p[order]
```

```
    adjusted = ranked * len(p) / np.arange(1, len(p) + 1)
    adjusted = np.minimum.accumulate(adjusted[::-1])[::-1]
    q = np.empty_like(adjusted)
    q[order] = np.minimum(adjusted, 1.0)
    result["BH_q_value"] = q
    return result


def ols_fit(use: pd.DataFrame, outcome: str, predictors: list[str])
-> dict:
    y = use[outcome].to_numpy(dtype=float)
    x = np.column_stack([np.ones(len(use)),
use[predictors].to_numpy(dtype=float)])
    beta = np.linalg.lstsq(x, y, rcond=None)[0]
    residual = y - x @ beta
    n, p = x.shape
    sigma2 = residual @ residual / (n - p)
    cov = sigma2 * np.linalg.inv(x.T @ x)
    se = np.sqrt(np.diag(cov))
    t_value = beta / se
    p_value = 2 * stats.t.sf(np.abs(t_value), df=n - p)
    critical = stats.t.ppf(0.975, df=n - p)
    ci = np.column_stack([beta - critical * se, beta + critical *
se])
    total = ((y - y.mean()) ** 2).sum()
    r2 = 1 - (residual @ residual) / total
    adj_r2 = 1 - (1 - r2) * (n - 1) / (n - p)
    return {"beta": beta, "ci": ci, "p": p_value, "r2": r2,
"adj_r2": adj_r2}


def logistic_fit(use: pd.DataFrame, outcome: str, predictors:
list[str]) -> dict:
    y = use[outcome].to_numpy(dtype=float)
    x = np.column_stack([np.ones(len(use)),
use[predictors].to_numpy(dtype=float)])

    def objective(beta):
        eta = x @ beta
        return float(np.logaddexp(0, eta).sum() - y @ eta)

    def gradient(beta):
        return x.T @ (expit(x @ beta) - y)

    fit = minimize(objective, np.zeros(x.shape[1]), jac=gradient,
method="BFGS",
                   options={"gtol": 1e-10, "maxiter": 1000})
    if not fit.success and np.linalg.norm(gradient(fit.x),
ord=np.inf) > 1e-6:
        raise RuntimeError(f"Logistic model did not converge:
{fit.message}")
    beta = fit.x
    probability = expit(x @ beta)
    information = x.T @ (x * (probability * (1 - probability))[:,
```

```
None])
    cov = np.linalg.inv(information)
    se = np.sqrt(np.diag(cov))
    z = beta / se
    p_value = 2 * stats.norm.sf(np.abs(z))
    ci = np.column_stack([beta - 1.959963984540054 * se,
                          beta + 1.959963984540054 * se])
    return {"beta": beta, "ci": ci, "p": p_value}


def fit_models(d: pd.DataFrame) -> pd.DataFrame:
    model_data = d.assign(GA=d["genotype"].eq("GA").astype(int))
    predictors = ["GA", "Varsta", "female", "Durata_boala_ani",
"biologic", "current_smoker"]
    records = []
    for outcome in ["ASDAS_CRP", "BASDAI"]:
        use = model_data[[outcome] + predictors].dropna()
        fit = ols_fit(use, outcome, predictors)
        for index, term in enumerate(predictors, start=1):
            records.append({
                "model": f"OLS {outcome}", "n": len(use), "term":
term,
                "estimate": fit["beta"][index], "ci_low": fit["ci"]
[index, 0],
                "ci_high": fit["ci"][index, 1], "p_value": fit["p"]
[index],
                "r_squared": fit["r2"], "adjusted_r_squared":
fit["adj_r2"],
            })
    use = model_data[["metabolic_syndrome"] +
predictors].dropna().copy()
    use["metabolic_syndrome"] =
use["metabolic_syndrome"].astype(int)
    fit = logistic_fit(use, "metabolic_syndrome", predictors)
    for index, term in enumerate(predictors, start=1):
        records.append({
            "model": "Logit metabolic syndrome", "n": len(use),
"term": term,
            "estimate": np.exp(fit["beta"][index]), "ci_low":
np.exp(fit["ci"][index, 0]),
            "ci_high": np.exp(fit["ci"][index, 1]), "p_value":
fit["p"][index],
            "r_squared": np.nan, "adjusted_r_squared": np.nan,
        })
    return pd.DataFrame(records)


def sensitivity_analysis(d: pd.DataFrame) -> pd.DataFrame:
    if "Waist_corrected" not in d.columns:
        raise ValueError(
            "Sensitivity analysis requires the non-identifying
Waist_corrected column"
        )
    corrected = d["Waist_corrected"].fillna(0).astype(int).eq(1)
```

```
    if corrected.sum() != 6:
        raise ValueError(f"Waist_corrected must identify exactly 6 
records; found {corrected.sum()}")
    subset = d.loc[~corrected].copy()
    rows = []
    for outcome in ["waist_high", "metabolic_syndrome"]:
        table = binary_genotype_table(subset, outcome)
        rows.append({"outcome": outcome, "n": len(subset),
                     "GA_n": int(table[0, 0]), "GA_total": 
int(table[0].sum()),
                     "GG_n": int(table[1, 0]), "GG_total": 
int(table[1].sum()),
                     **conditional_or(table)})
    return pd.DataFrame(rows)


def sex_specific_basdai(d: pd.DataFrame) -> pd.DataFrame:
    rows = []
    for sex in ["M", "F"]:
        gg = d.loc[(d["Gen"] == sex) & (d["genotype"] == "GG"), 
"BASDAI"].dropna()
        ga = d.loc[(d["Gen"] == sex) & (d["genotype"] == "GA"), 
"BASDAI"].dropna()
        test = stats.ttest_ind(gg, ga, equal_var=False)
        rows.append({"sex": sex, "GG_n": len(gg), "GG_mean": 
gg.mean(), "GG_sd": gg.std(ddof=1),
                     "GA_n": len(ga), "GA_mean": ga.mean(), "GA_sd": 
ga.std(ddof=1),
                     "welch_p": test.pvalue})
    return pd.DataFrame(rows)


def write_summary(d: pd.DataFrame, outdir: Path) -> None:
    gg = int((d["genotype"] == "GG").sum())
    ga = int((d["genotype"] == "GA").sum())
    aa = 0
    met_alleles = ga + 2 * aa
    total_alleles = 2 * len(d)
    pearson_r, pearson_p = stats.pearsonr(d["BMI"], 
d["Circumf_abd_cm"])
    slope, intercept, lin_r, lin_p, stderr = 
stats.linregress(d["BMI"], d["Circumf_abd_cm"])
    lines = [
        f"N = {len(d)}", f"GG = {gg}; GA = {ga}; AA = {aa}",
        f"Met allele frequency = {met_alleles / total_alleles:.6f}",
        f"HWE exact p = {hwe_exact(ga, gg, aa):.6f}",
        f"Harmonized waist criterion = {int(d['waist_high'].sum())}/
{len(d)}",
        f"Harmonized metabolic syndrome = 
{int(d['metabolic_syndrome'].sum())}/{len(d)}",
        f"NCEP waist sensitivity = 
{int(d['waist_high_ncep'].sum())}/{len(d)}",
        f"NCEP metabolic syndrome sensitivity = 
{int(d['metabolic_syndrome_ncep'].sum())}/{len(d)}",
```

```
        f"BMI-waist Pearson r = {pearson_r:.6f}; p =
{pearson_p:.8g}",
        f"BMI-waist slope = {slope:.6f} cm per BMI unit; intercept =
{intercept:.6f}",
    ]
    for sex, label in [("M", "Men"), ("F", "Women")]:
        values = d.loc[d["Gen"] == sex, "Circumf_abd_cm"]
        lines.append(f"{label} waist = {values.mean():.6f} ±
{values.std(ddof=1):.6f} cm")
    (outdir / "analysis_summary.txt").write_text("\n".join(lines) +
"\n", encoding="utf-8")


def main() -> None:
    args = parse_args()
    args.output_dir.mkdir(parents=True, exist_ok=True)
    d = derive_variables(read_dataset(args.input, args.sheet,
args.header_row))
    write_summary(d, args.output_dir)
    genotype_binary_results(d).to_csv(args.output_dir /
"genotype_binary_results.csv", index=False)
    sensitivity_analysis(d).to_csv(args.output_dir /
"waist_sensitivity_analysis.csv", index=False)
    correlations(d).to_csv(args.output_dir /
"spearman_correlations_with_BH.csv", index=False)
    fit_models(d).to_csv(args.output_dir /
"adjusted_regression_models.csv", index=False)
    sex_specific_basdai(d).to_csv(args.output_dir /
"sex_specific_BASDAI.csv", index=False)


if __name__ == "__main__":
    main()
```
